# Nursing students’ perceptions of the use of clinical simulation in biosafety education

**DOI:** 10.1590/1980-220X-REEUSP-2025-0116en

**Published:** 2026-09-14

**Authors:** Bruna Pedroso Canever, Saionara Nunes de Oliveira, Caroline Cechinel Peiter, Samara Eliane Rabelo Suplici, Diana Coelho Gomes, Maria Lígia dos Reis Bellaguarda, Diovane Ghignatti da Costa, Aline Lima Pestana Magalhães, Fernanda Marques de Oliveira

**Affiliations:** 1 Universidade Federal de Santa Catarina, Departamento de Enfermagem, Graduação em Enfermagem, Florianópolis, SC, Brazil.; 2 Hospital Universitário Professor Polydoro Ernani de São Thiago, Florianópolis, SC, Brazil.; 3 Unidade Local de Saúde de Santa Maria, Serviço de Pneumologia - Internamento Geral, Lisboa, Portugal.

**Keywords:** Simulation Training, Learning, Patient Safety, Containment of Biohazards, Education, Nursing

## Abstract

**Objective:**

To understand nursing students’ perceptions of the use of clinical simulation in biosafety education.

**Method:**

A qualitative, exploratory, and descriptive study conducted in 2023 based on the implementation of clinical simulation for biosafety learning. Participants were 26 students enrolled in the “Fundamentals of Nursing” course of an undergraduate nursing program at a federal university in southern Brazil. Data were analyzed using IRAMUTEQ^®^ software.

**Results:**

Three categories emerged: (1) Emotional regulation for interaction and communication with patients and families, highlighting feelings of anxiety and the need for emotional regulation during the application of standard precautions; (2) Contributions of simulation to learning biosafety practices, emphasizing a better understanding of the use of personal protective equipment, risk perception, and the integration of theory and practice; and (3) Strengths and weaknesses perceived by students regarding their performance, including self-assessment, recognition of technical limitations, and strengthened teamwork.

**Conclusion:**

Clinical simulation was perceived as a strategy that promotes learning of biosafety practices by integrating technical, emotional, and communication-related aspects, thereby contributing to safer and more reflective professional education.

## INTRODUCTION

The development of competencies and knowledge in biosafety is fundamental to nursing education, promoting behavioral change, good healthcare practices, and patient safety. The basic principles of biosafety must be firmly established so that students are able to understand situations requiring the application of this knowledge throughout their educational journey. In this context, standard precautions constitute one of the fundamental principles of biosafety, encompassing the set of measures that must be applied when providing care to all patients^(1)^.

Standard precautions should be implemented in every healthcare setting to prevent healthcare-associated infections (HAIs). HAIs represent a major public health problem, threatening patients’ lives, prolonging hospital stays, and increasing healthcare costs^(2)^. Standard precautions include hand hygiene, the use of personal protective equipment (PPE) appropriate to the type of exposure, proper disposal of contaminated materials, implementation of respiratory hygiene/cough etiquette, patient isolation, and cleaning and disinfection of surfaces^(3)^.

Despite the relevance of this topic, studies have shown that nursing students’ and professionals’ knowledge regarding standard precautions remains insufficient^(4)^. Consequently, educators and researchers in the health field have expressed concern about how biosafety is taught, particularly in the current context, in which teaching and learning processes have undergone substantial changes^(5)^.

Accordingly, there is a need to incorporate educational strategies that promote meaningful learning, are aligned with social realities, and effectively encourage the adoption of good healthcare practices^(6)^. For this reason, understanding students’ perceptions of different teaching approaches is essential, as it promotes their active participation and encourages greater engagement in constructing their own learning process^(7)^.

Within this context, active learning methodologies transcend the traditional teaching model, in which the instructor transmits knowledge and serves as the central source of information. Instead, students become the central agents in the learning process, playing an active role and responsibility for seeking and analyzing knowledge. Thus, active learning methodologies foster students’ critical, creative, and reflective thinking, both individually and collectively, by encouraging them to identify different approaches to solving problems^(5)^.

Among the various active learning methodologies, clinical simulation stands out as a strategy that creates a safe and controlled environment in which participants actively engage in the learning process by practicing, analyzing, and making decisions based on a problem-based scenario. In this way, simulation follows predetermined phases for knowledge acquisition and guides the decision-making process^(8)^.

Each phase enables students to identify problems, establish diagnoses, and determine appropriate solutions. Knowledge acquisition occurs when students express their thoughts and feelings, report doubts, uncertainties, and limitations in their ability to act and self-assess, learning from both their own experiences and those of their peers^(8)^.

Experiencing situations that require the application of biosafety knowledge in a safe environment, without posing risks to patients or healthcare professionals/students, may be an effective way of translating previously acquired theoretical knowledge into practice^(6)^. From this perspective, simulation as a learning strategy promotes the development of more consistent knowledge, skills, and attitudes, thereby contributing to the quality of nursing care^(9)^.

Although recent literature has demonstrated the benefits of clinical simulation in healthcare education—including improvements in knowledge, clinical competence, and self-confidence^(10,11)^—studies exploring its application specifically in biosafety education, and particularly students’ perceptions of this pedagogical strategy in this context, remain scarce^(12,13)^. Considering the shortcomings already identified regarding adherence to standard precautions and infection prevention and control, it is important to determine whether clinical simulation is a valuable tool for addressing educational gaps related to patient safety and safe professional practice.

Given the above, the following research question was formulated: How do nursing students perceive the use of clinical simulation as a teaching tool for biosafety? Therefore, this study aimed to understand nursing students’ perceptions of the use of clinical simulation in biosafety education.

## METHOD

### Ethical Aspects

This study was submitted to and approved under Certificate of Presentation for Ethical Consideration 66654622.0.0000.0121 and by the Research Ethics Committee involving Human Beings, under Opinion 6,021,025. The study complied with the ethical principles and regulatory guidelines established by Resolution 466/2012 of the Brazilian National Health Council.

Participants signed the Informed Consent Form in person before beginning the research activity. Participants were identified using the initial letter “e” followed by a sequential number (e01, e02, e03...) to preserve their anonymity.

### Study Design

This is a descriptive qualitative study based on the International Nursing Association for Clinical Simulation and Learning (INACSL) theoretical-methodological framework^(14)^, which provides best practice standards for the development of clinical simulation. The methodological organization of this study followed COnsolidated criteria for REporting Qualitative research recommendations^(15)^.

### Methodological Procedures

Students were invited to participate through a direct invitation in the classroom, during which the study objectives and procedures were explained. Students aged 18 years or older who were enrolled in the “Fundamentals of Nursing” course, offered during the third semester of the undergraduate nursing program, and who were available to participate in the simulation outside regular class hours were eligible for inclusion. It should be noted that all students had already attended the theoretical class on biosafety and standard precautions before participating in the simulated practice.

Before the simulation, participants completed a characterization instrument developed by the researchers, which included information such as age, sex, previous experience with clinical simulation, and professional experience in the healthcare field. Students who had not attended the theoretical class on biosafety were excluded.

Data were collected using a self-administered questionnaire composed exclusively of open-ended questions, developed to explore participants’ perceptions regarding the learning, knowledge, attitudes, clinical reasoning, and critical thinking developed through the simulation practice. The use of a self-administered instrument was intended to encourage the free expression of individual experiences while minimizing researcher interference during the data generation process.

### Study Setting

The simulation practice laboratory of the undergraduate nursing program (LABEnf) at a federal public university in southern Brazil served as the setting for this study.

### Data Collection and Organization

Before data collection began, a pilot test of the simulation scenario was conducted with students from other semesters of the undergraduate nursing program. The purpose was to assess the clarity of the instructions, the time required to complete the activity, and the organization of the resources used. Data obtained during this stage were not included in the analysis. The pilot test allowed minor adjustments to be made to the organization of the scenario without modifying its objectives.

Data were collected during April, May, and August 2023. To administer the self-administered questionnaire, a clinical simulation was designed in which students participated in an immersive simulation experience before completing the research instrument. The simulation was organized according to the INACSL Standards of Best Practice^(14)^, following the stages described in Chart 1.

**Chart 1:** Stages of conducting the clinical simulation – Florianópolis, SC, Brazil, 2023.

**Planning:** The choice of the biosafety theme was based on difficulties identified by students and faculty during the theoretical-practical activities of the “Fundamentals of Nursing” course. At this stage, the facilitator responsible for conducting the simulation was designated.	
**Learning objectives:** **General objective** Enable students to properly use personal protective equipment when caring for patients, taking standard precautions into account. **Specific objectives** Correctly use personal protective equipment in accordance with standard precautions; Identify risks of biological exposure; Adopt biosafety measures during patient care; Implement appropriate actions in response to clinical complications. The objectives were presented to participants during the briefing.	
**Expected conduct from participants:** The expected conduct corresponds to the observable behaviors used to assess the achievement of the proposed objectives. – Hand hygiene at the appropriate times; – Proper use of personal protective equipment (as required); – Safe handling of secretions and body fluids; – Appropriate conduct following exposure to biological material; – Effective communication with the patient and their companion.	
**Simulation structure:** The clinical simulation was conducted with students organized into randomly assigned pairs. Each pair actively participated in the scenario. After completing the scenario, students took on the role of observers for the subsequent pairs. Stage duration: **Briefing:** 10 minutes **Simulation scene:** 15 minutes **Debriefing:** 30 minutes	
**Setting and environment organization** The simulation took place in an environment designed to replicate a hospital medical ward room with two occupied beds, allowing participants to experience simultaneous care situations. Each bed was equipped with a hospital bed, linens, and a headboard set to a Fowler position, adjusted according to the simulated clinical condition. Bedside tables held patient charts containing clinical information. Care supplies were organized on an easily accessible side counter. Trained actors portrayed patients, supported by an arm simulator for venipuncture, which enhanced the realism of the procedures. Personal protective equipment was made available in a visible and accessible location, encouraging its use in accordance with standard precautions. The environment was organized to ensure clinical fidelity, fostering participant immersion and the execution of expected clinical actions.	
**Materials and resources:** Materials compatible with the simulated clinical environment were used, including: Personal protective equipment; Supplies for handling biological material; Materials for hemorrhage control; Venipuncture simulation device.	
**Description of clinical cases**	
**Clinical case 1:** Maria, 24, was admitted to the medical ward with a diagnosis of pneumonia. She reported feeling feverish and experiencing chills since early morning. The patient presented with nausea and episodes of vomiting; she kept a container of gastric contents by her side, which was handed to the healthcare professional during the assessment. She had peripheral venous access and was accompanied by her cousin.	**Clinical case 2:** Paula, 22, was admitted to the medical ward with a diagnosis of pyelonephritis. She has a peripheral IV line in place and is receiving antibiotic therapy. She experiences a bout of renal colic while the nurse is attending to the patient in the adjacent bed; she inadvertently jerks her arm, dislodging the IV line. Bleeding begins and requires control. Her companion calls for help.
**Briefing:** The briefing lasted approximately 10 minutes and was conducted immediately before the beginning of the simulation. During this stage, participants received general information about the activity, including the objectives of the simulation, the scenario context, the time allocated for the activity, and the resources available in the environment. Following this initial orientation, the standardized instructions were read to students, as described below: **Instructions provided to participants** Students were instructed to play the role of two nurses responsible for patients admitted to a hospital medical ward room with two occupied beds. They were informed that they had just received the shift handover and should jointly begin the assessment of both patients. Participants were also informed that clinical complications could occur during patient care, requiring decision-making, prioritization of care, and teamwork. They were expected to provide care collaboratively, taking full responsibility for the patients’ care, including clinical assessment, performance of procedures, and management of potential complications. The importance of implementing biosafety measures in all situations was emphasized, including the appropriate use of personal protective equipment, particularly when there was a risk of exposure to body fluids. Furthermore, participants were informed that, throughout the simulation, they should: Assess patients’ clinical conditions; Identify high-risk situations; Intervene in a timely manner; Organize care simultaneously for both patients in the same setting. Finally, it was announced that the simulation would last 15 minutes, with the start and end times signaled by the facilitation team. It was also stated that the scenario provided all necessary materials for patient care—including personal protective equipment, supplies for managing secretions and controlling bleeding, and equipment for venous access—which could be used at participants’ discretion.	
**Debriefing:** Conducted immediately after the simulation and lasting approximately 30 minutes, it employed the Gather-Analyze-Summarize technique^(5)^. Participants in this stage included the facilitator and a faculty member who acted as the patient in the scenario. The following guiding questions were used: a) How did you feel during the simulation? b) How do you assess your performance during the scene? c) What were your main strengths? d) What aspects could be improved, and how would you improve them in a future situation??	
**Assessment:** The assessment was integrated into the debriefing process through the participants’ reflective self-assessment, considering technical and behavioral aspects as well as the adoption of biosafety measures. Pre-defined expected actions served as the reference for performance analysis, facilitating the identification of strengths, gaps, and opportunities to enhance clinical competencies.	

Data were collected using a self-administered questionnaire consisting of four open-ended questions, developed to explore participants’ perceptions of the clinical simulation. The instrument was administered immediately after the debriefing. It should be noted that the time allocated for the debriefing was separate from the time allotted for completion of the questionnaire, the latter lasting approximately 25 to 30 minutes.

### Data Analysis

Data were organized in a Microsoft Office Excel^®^ spreadsheet containing participants’ demographic characteristics and responses to the qualitative study questions. Subsequently, the textual data were organized in an OpenOffice^®^ document and reviewed by a second researcher to eliminate typographical and spelling errors and to standardize terminology and abbreviations. The corpus was then analyzed using *Interface de R pour les Analyses Multidimensionnelles de Textes et de Questionnaires* (IRAMUTEQ^®^), version 0.7 alpha 2. Classical textual statistical analyses and Descending Hierarchical Classification (DHC) were performed. DHC groups text segments into lexically homogeneous classes based on word frequency and co-occurrence, represented in a dendrogram. The following word classes were included in the analysis: adjectives, nouns, verbs, and forms not recognized by the IRAMUTEQ^®^ dictionary.

The resulting classes were interpreted qualitatively based on content analysis^(16)^, relating the identified lexical nuclei to the study objectives and to the perceptions expressed by participants. The interpretation of the classes was carried out carefully by the researchers to identify representative thematic categories and ensure consistency between the lexical findings and the discussion of results.

## RESULTS

Of the 65 students enrolled during the two academic semesters in which data collection was conducted, 41 met the study inclusion criteria, of whom 26 attended the simulation activity and were included in the study.

Among the 26 participants, most were female (23; 89%). Participants’ ages ranged from 20 to 24 years. None of the participants had previous experience with clinical simulation or worked in the healthcare field. Textual data analysis showed that the corpus consisted of 182 text segments and 3,981 occurrences, comprising 708 distinct forms. The mean number of words per text segment was 22. Classical textual statistical analysis identified “patient”, “situation”, “simulation”, “to_manage”, “calm”, and “to_feel” as the most frequent terms.

**Figure 1:**
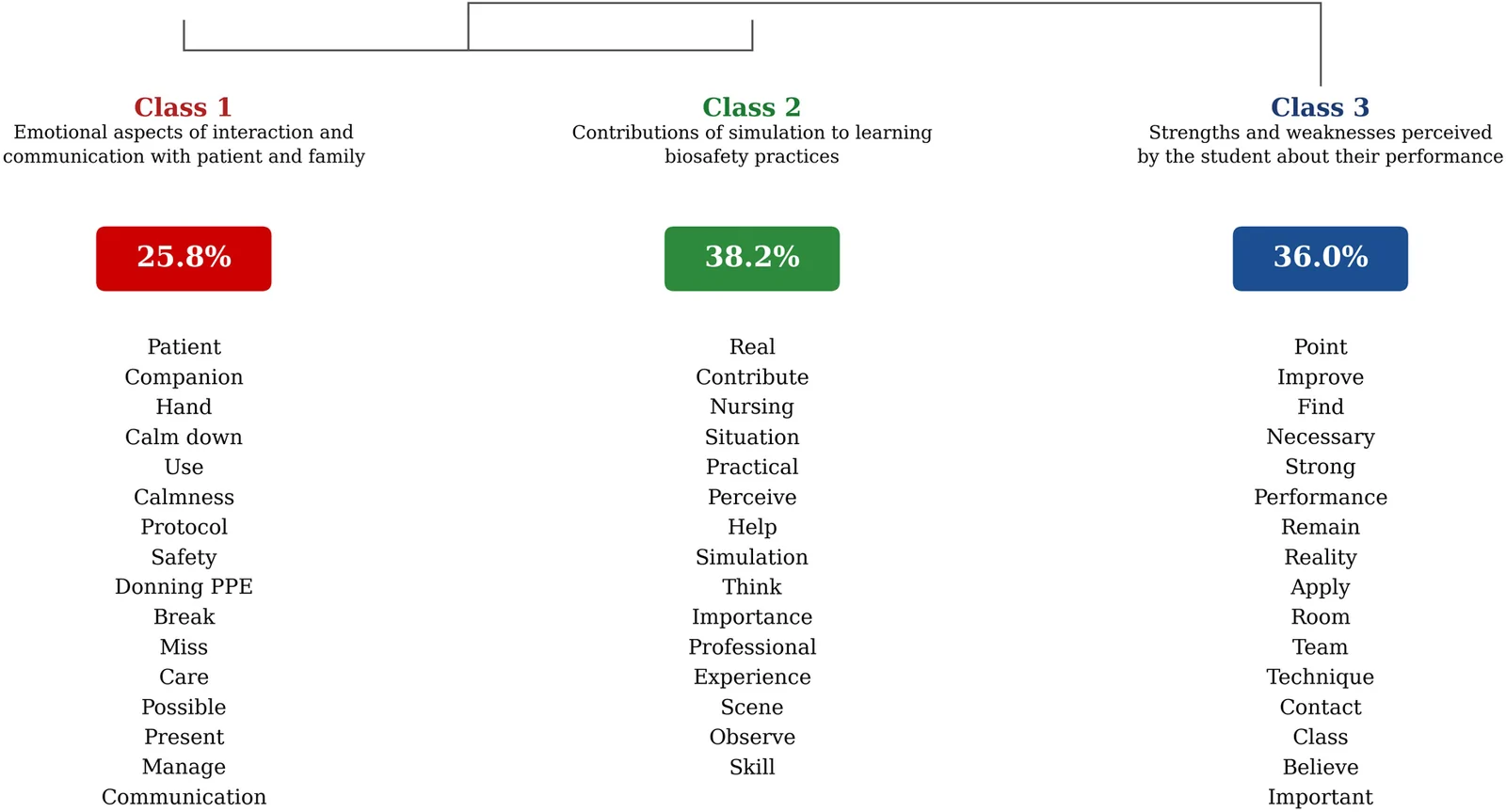
Descending Hierarchical Classification performed using IRAMUTEQ^®^ software – Florianópolis, SC, Brazil, 2025.

Based on the DHC, the following semantic classes were identified: (1) Emotional regulation for interaction and communication with patients and families; (2) Contributions of simulation to learning biosafety practices; and (3) Strengths and weaknesses perceived by students regarding their performance. The DHC performed using IRAMUTEQ^®^ showed that classes 2 and 3 were related to each other and were hierarchically associated with class 1 (Figure 1).

Class 1 (“Emotional regulation for interaction and communication with patients and families”) revealed that participants recognized the importance of regulating their emotions during patient encounters and the impact of this emotional regulation on effective communication with patients and their accompanying family members. Students also recognized the importance of remaining calm while providing care, as this influenced the confidence of both patients and their accompanying family members, strengthened the therapeutic relationship, and contributed to patient safety.

*Calming the patient and their companion so that care can be provided more calmly, thereby helping to reduce errors—such as improper use of PPE—that might arise from the restlessness of both parties* (e14).

*Maintaining my own composure so that I can then calm the patient and/or their companion* (e11).

*Calming the companion and the patient without becoming flustered* (e18).

*Staying calm helps in maintaining control over the environment and the situation* (e19).

*I realized the importance of adaptability and emotional regulation in nursing* (e19).

However, despite recognizing the importance of communication with patients and their accompanying family members, as well as their active participation during the simulation, many students reported feeling nervous, anxious, fearful, and tense when interacting with patients, which often affected their performance during the simulation scenario.

*I tried to calm her down but couldn’t. I asked when she had been admitted to the hospital and about her pain level, but only because I had help; in my nervousness, I hadn’t remembered to do so* (e05).

*Calming the companion and the patient without getting flustered, and realizing I need to improve at working under pressure* (e18).

*Feeling nervous about having to talk to a patient and not knowing how to answer his questions* (e19).

Participants expressed feelings of empathy and a strong desire to help patients. However, students’ emotional distress was largely driven by concern for patients.

*Driven by the urge to help the patient, I failed to coordinate the care with safety protocols* (e01).

*I realized how lost I was; I feared the patient would be in pain* (e04).

*An attempt to listen to the patient’s needs and help as quickly as possible* (e09).

*Frustrated and distressed. A sense of powerlessness regarding patient care* [...] *and fear of being unable to perform the procedures due to a lack of practice* (e09).

*A mix of nervousness and fear of being unable to perform the necessary procedures, alongside the desire to help the patient in need* (e10).

Class 2 (“Contributions of simulation to learning biosafety practices”) showed that students perceived several contributions of clinical simulation to the development of skills and competencies related to biosafety, particularly in strengthening their understanding of donning procedures and the appropriate use of PPE, both of which are fundamental components of standard precautions.

*This simulation helps us understand the risks we and our patients face when we do not prioritize biosafety* (e13).

*Through the simulation, I was able to identify practical nursing skills that I need to improve for my daily practice—such as using a mask when interacting with a patient who has symptoms and a diagnosis of a respiratory infection* (e08).

*It helped prepare me for unexpected situations by highlighting essential actions*—[for instance], *using PPE in a scenario where I need to act quickly* (e26).

*It served as a reminder of the importance of paying attention to PPE and how crucial it is to focus on biosafety, whether regarding oneself or the patient* (e15).

Based on the simulation experience, participants reported that the clinical simulation on biosafety provided an experience that closely reflected nurses’ day-to-day practice and better prepared them for real-world clinical situations—that is, for what they will encounter in clinical practice—thereby strengthening the connection between theory and practice. Students also reported a better understanding of the techniques, greater assimilation of the theoretical content, recognition of areas requiring further development, increased experience, and greater exposure to situations requiring decision-making related to biosafety practices.

*Seeing a real-life case makes us reflect and visualize how to act and what to do in practice* (e10).

*Now I know what I need to improve, and I view this experience as an important step in my professional development* (e21).

*As soon as I started my shift, I immediately considered the risk of contamination and thought about how to organize the work: putting on gloves, staying calm, verifying patient details, and avoiding the incorrect use of masks* (e22).

*It aids the learning process and helps us master biosafety techniques that are essential for our health* (e25).

*Even though I wasn’t able to handle the scene exactly as I would have liked, I think it was a valuable learning experience. I won’t repeat the same mistakes, and I won’t forget the importance of wearing gloves* (e18).

In addition to technical skills, students also perceived that the simulation fostered the development of emotional competencies. They reported feelings of achievement, self-confidence, and confidence in performing the techniques, leading them to recognize the importance of clinical simulation for learning.

*I felt pressured by the situation involving two patients who needed my help, and there was barely enough time to put on my protective gear. However, looking back, I’m happy I managed to resolve the problem* (e06).

*The highlight for me was correctly performing the techniques according to established protocols, which gave me more confidence to handle real-life situations* (e11).

*The simulation helped me realize the importance of staying calm; while there will be moments that feel desperate, we have trained for this, and by remaining calm, we can recall the necessary steps to help the patient without putting our own health at risk* (e25).

*It was challenging but highly enriching* (e17).

*I started the simulation feeling very anxious and afraid of making mistakes, but my confidence grew as I performed the techniques* (e26).

In addition, students emphasized the importance of observing their peers during the simulation scenario, as well as the debriefing session as an opportunity for reflection and learning.

*Seeing my colleagues helped me realize things I could have done but hadn’t thought of in the heat of the moment* (e11).

*It helped put our knowledge into practice, and watching colleagues perform allowed me to notice things I had overlooked* (e18).

*It was only after it ended and we sat in the circle that I was able to reflect on the challenges faced and realize how much this experience helped me develop new skills* (e22).

*It helped by providing a chance to practice and making me feel comfortable making mistakes, since it was just a simulation. The debriefing session aided in the group-based development of clinical reasoning* (e26).

Class 3 (“Strengths and weaknesses perceived by students regarding their performance”) showed that the main weaknesses identified by students were related to communication skills and emotional regulation.

*Improve communication, use work materials more appropriately, and practice hand hygiene before coming into contact with patients* (e03).

*I need to improve my concentration and attention, and better manage my nervousness and anxiety, as well as avoid getting tense about the whole situation* (e13).

*I need to work on managing my anxiety better* (e11).

*It went smoothly for me. I think I managed to do what was required, but I couldn’t finish the scene because my patient asked a lot of questions and I didn’t know how to extricate myself from the conversation* (e23).

Some participants reported feeling calm and confident when performing biosafety techniques, representing an important strength identified in their performance. These findings suggest that when emotional regulation is maintained, students’ performance is positively influenced, thereby contributing to both patient and healthcare worker safety.

*I believe one of the strengths of my performance was my patience and adherence to standard precautions regarding glove use when coming into contact with the patient’s blood* (e08).

*I believe my performance during the simulation had several strengths. I managed to stay calm and safely apply the knowledge I had gained in the classroom* (e12).

*I was able to apply classroom knowledge to a real-world scenario, but in a safer, controlled manner that allowed me to test things and receive feedback without posing risks to the patients or ourselves* (e17).

*All sorts of information was racing through my mind; I had to focus to hear the patient’s account amidst the screaming, all while remembering to ensure both my safety and the patient’s* (e02).

*I successfully performed the techniques according to protocol and remained calm throughout the entire process* (e11).

Another strength identified in students’ performance during the clinical simulation was their ability to work as a team. When communication was clear and effective, students perceived that care was delivered with greater organization, efficiency, quality, and safety.

*One of my key strengths was the ability to work as part of a team* (e13).

*My approach focused heavily on communication with the team. I tried to maintain a good dialogue with my colleagues to ensure everyone was aligned*. [...] *I maintained clear communication with my colleague. This helped prevent errors and made the care process more efficient and organized* (e16).

*My strength was being proactive in communicating with my partner, ensuring a collaborative and organized environment* (e20).

*My partner and I were quick to react, each moving to attend to the patient* (e02).

Based on the strengths and weaknesses identified through the clinical simulation, participants recognized the need for a deeper understanding of the theoretical and practical content, highlighting this as an important area for improvement. Through the clinical simulation, students were able to identify which biosafety-related techniques require further development.

*Improve technique by attending tutoring sessions* (e21).

*Gain a deeper understanding of what we learned in the classroom* (e24).

*I think improving the technical aspect* (e25).

*The ability to gown up more quickly and perform proper asepsis, even when time is limited* (e09).

*Gowning up, in general, needs to be done with greater precision, in addition to knowing what to use at each stage* (e10).

## DISCUSSION

Class 1 demonstrated that the emotional dimension was central to students’ experience. Feelings such as nervousness, fear, insecurity, and anxiety were reported during the simulated activities, particularly while being observed by peers and faculty members, a finding that has also been reported in previous studies^(17,18)^. These findings suggest that simulation elicits emotional responses similar to those experienced in real clinical settings, bringing students closer to the demands of clinical practice.

However, these emotions were not perceived solely as barriers but also as integral components of the learning process. The psychologically safe simulation environment allowed students to experience these emotions without placing patients at risk, enabling them to be explored through structured debriefing. Post-simulation reflection facilitated understanding of the decisions made, identification of errors, and modification of subsequent actions^(19)^, thereby strengthening critical thinking and students’ ability to act more confidently.

In the context of biosafety, the emotional dimension plays a particularly important role. Adherence to standard precautions requires not only technical knowledge but also the ability to maintain safe practices in situations involving pressure or uncertainty. Recent evidence indicates that high levels of stress may interfere with nursing students’ decision-making and clinical performance, reinforcing the importance of developing emotional self-regulation to support safe practice^(20)^.

Communication also emerged as an essential aspect of the simulation experience. Students reported both advances and challenges in their interactions with patients, family members, and the healthcare team. Effective communication is a fundamental component of patient safety because it facilitates the clear exchange of information and the coordination of care^(21,22)^. Thus, clinical simulation proved to be a valuable environment for the integrated development of communication skills and biosafety practices, extending learning beyond the acquisition of technical skills.

Class 2 demonstrated that clinical simulation contributed to consolidating learning in biosafety, particularly by promoting the integration of theory and practice in contextualized situations. Beyond technical performance, students reported greater awareness of the biological risks associated with patient care and a better understanding of the application of standard precautions and the appropriate use of PPE. Their ability to anticipate the risk of contamination and to plan appropriate actions in advance reflects the development of clinical reasoning focused on safety.

Reviews of biosafety education in Brazil indicate that this topic is still frequently taught in a fragmented manner and primarily through traditional teaching approaches^(23)^. In this context, clinical simulation proved capable of integrating technical knowledge, clinical judgment, and professional responsibility within a single scenario, bringing students closer to the real demands of clinical practice.

The findings of this study are consistent with evidence showing that simulation-based educational interventions promote critical-reflective learning by allowing students to identify errors and recognize shortcomings in a safe environment^(24,25)^. However, unlike exclusively lecture-based approaches, the simulated experience requires decision-making under pressure and the active application of knowledge, thereby narrowing the gap between knowing and doing.

Another relevant aspect concerns learning through errors. By recognizing mistakes in the performance of the techniques and expressing their intention not to repeat them, students demonstrated internalization of learning, reinforcing the importance of a safe environment for experimentation and the refinement of clinical practice.

Previous experience in a controlled environment was also associated with increased self-confidence and a greater sense of preparedness for clinical practice^(26,27)^. This finding is particularly relevant in biosafety education, as adherence to preventive measures depends not only on knowledge of the protocols but also on the ability to apply them safely in urgent and unpredictable situations. Thus, clinical simulation contributes to strengthening safe practice and protecting both patients and healthcare professionals.

Class 3 revealed that clinical simulation promoted processes of self-assessment and recognition of individual weaknesses. Students identified limitations mainly related to communication, decision-making, and the technical performance of biosafety practices, such as proper donning of PPE, aseptic technique, and anxiety management in high-pressure situations. This recognition demonstrates that the experience enabled students to critically reflect on their own performance.

This reflective process highlights the role of clinical simulation as a strategy that promotes self-reflection and professional development. The opportunity to make mistakes in a safe environment, receive feedback, and reconsider one’s actions contributes to the development of a more critical and responsible professional attitude^(28)^. In the context of biosafety, this process is particularly important because adherence to preventive measures requires not only knowledge of the protocols but also continuous attention to how they are applied.

The ability to work as part of a team was also perceived as an important strength. When communication was clear and effective, students reported greater organization and efficiency in the delivery of care. These findings reinforce that safe biosafety practices depend not only on the correct individual performance of technical procedures but also on effective collaboration among team members, as interventions aimed at improving teamwork have been associated with enhanced patient safety and reduced healthcare errors^(29)^.

In addition, prior preparation for complex situations before entering the clinical setting was identified as another positive aspect. High-fidelity simulation enables students to experience situations that closely resemble real clinical practice without exposing patients or learners to actual risks^(17,28)^, thereby promoting greater confidence and preparedness for future clinical practice.

When the three classes are considered together, the findings indicate that clinical simulation contributes to biosafety education in a multidimensional manner. In addition to knowledge acquisition, the simulated experience promotes emotional development, enhances communication skills, consolidates theoretical knowledge, and fosters self-reflection.

These findings reinforce that biosafety education, when supported by experiential learning methodologies, can contribute to the preparation of healthcare professionals who are more confident, critical, and better prepared to deal with situations involving biological risks. For educators, the findings highlight the importance of designing simulation scenarios that integrate both technical and relational dimensions, thereby promoting comprehensive nursing education.

### Study Limitations

A limitation of this study is that it was conducted at a single educational institution, which may limit the generalizability of the findings to other educational settings.

### Contributions to Nursing

This study contributes to biosafety education in undergraduate nursing by demonstrating that clinical simulation promotes not only the consolidation of technical knowledge related to standard precautions but also the development of emotional, communication, and reflective competencies that are essential for safe clinical practice.

The findings indicate that the structured use of simulation scenarios can prepare students for entry into clinical settings by strengthening self-confidence, decision-making, and commitment to patient safety. For nurse educators, the results reinforce the importance of incorporating simulation activities, combined with structured debriefing sessions, into educational planning to promote critical reflection on biosafety practices and emotional management in clinical care situations.

## CONCLUSION

This study showed that, from the perspective of nursing students, clinical simulation is a valuable strategy for biosafety education, as it promotes the integration of theory and practice, strengthens self-confidence, and fosters the development of technical and reflective competencies. Participants recognized that the simulated experience contributed to greater confidence in the application of standard precautions while also highlighting challenges related to emotional regulation and communication skills in situations requiring immediate decision-making.

The strengths identified, such as teamwork and the ability to recognize areas for improvement, indicate that simulation supports self-assessment and professional development. Thus, beyond technical instruction, clinical simulation proved to be a valuable educational strategy in nursing education, particularly with regard to safe practice and patient safety. Future studies are recommended to investigate the longitudinal impact of simulation on adherence to biosafety practices in clinical settings and to explore its application across different institutional contexts.

## Data Availability

The entire dataset supporting the results of this study was published in the article itself.
