# Supplementary material for: Nursing students’ perceptions of the use of clinical simulation in biosafety education
Source: Rev Esc Enferm USP. 2026 Sep 14;60:e20250116. doi: 10.1590/1980-220X-REEUSP-2025-0116en (PMC13577235; doi:10.1590/1980-220X-REEUSP-2025-0116en)
Supplement: Translation

# Translation: Percepção de estudantes de enfermagem sobre a utilização da simulação clínica no ensino de biossegurança

**DOI:** 10.1590/1980-220X-REEUSP-2025-0116pt

**Authors:** Bruna Pedroso Canever, Saionara Nunes de Oliveira, Caroline Cechinel Peiter, Samara Eliane Rabelo Suplici, Diana Coelho Gomes, Maria Lígia dos Reis Bellaguarda, Diovane Ghignatti da Costa, Aline Lima Pestana Magalhães, Fernanda Marques de Oliveira

**Affiliations:** 1 Universidade Federal de Santa Catarina, Departamento de Enfermagem, Graduação em Enfermagem, Florianópolis, SC, Brasil.; 2 Hospital Universitário Professor Polydoro Ernani de São Thiago, Florianópolis, SC, Brasil.; 3 Unidade Local de Saúde de Santa Maria, Serviço de Pneumologia - Internamento Geral, Lisboa, Portugal.

**Keywords:** Treinamento por Simulação, Aprendizagem, Segurança do Paciente, Contenção de Riscos Biológicos, Educação em Enfermagem

## Abstract

**Objetivo:**

Compreender a percepção de estudantes de enfermagem acerca da utilização da simulação clínica no ensino de biossegurança.

**Método:**

Estudo qualitativo, exploratório e descritivo, desenvolvido em 2023 a partir da aplicação da simulação clínica para o aprendizado em biossegurança. Participaram 26 estudantes da disciplina “Fundamentos de Enfermagem” de um curso de graduação de uma universidade federal do Sul do Brasil. Os dados foram analisados com auxílio do *software* IRAMUTEQ^®^.

**Resultados:**

Emergiram três categorias: (1) Domínio emocional para a interação e comunicação com o paciente e família, evidenciando sentimentos de ansiedade e necessidade de controle emocional durante a aplicação das precauções padrão; (2) Contribuições da simulação para o aprendizado das práticas de biossegurança, destacando maior compreensão do uso de equipamentos de proteção individual, percepção de risco e articulação entre teoria e prática; e (3) Potencialidades e fragilidades percebidas pelo estudante sobre sua atuação, incluindo autoavaliação, reconhecimento de limitações técnicas e fortalecimento do trabalho em equipe.

**Conclusão:**

A simulação clínica foi percebida como estratégia que favorece o aprendizado das práticas de biossegurança ao integrar aspectos técnicos, emocionais e comunicacionais, contribuindo para uma formação mais segura e reflexiva.

## INTRODUÇÃO

O desenvolvimento de competências e conhecimentos em biossegurança é fundamental para a formação profissional em enfermagem, para a mudança de comportamento, para a promoção de boas práticas em saúde e para a segurança do paciente. Os princípios básicos de biossegurança precisam estar consolidados para que os estudantes consigam compreender as situações que exijam a aplicabilidade desses conhecimentos ao longo da sua jornada formativa. Nesse sentido, a precaução padrão é um dos princípios básicos da biossegurança, compreendendo o conjunto de medidas que devem ser aplicadas no atendimento a todos os pacientes^(1)^.

As medidas de precaução padrão devem ser empregadas em todo ambiente de cuidado para prevenir as infecções relacionadas à assistência à saúde (IRAS). As IRAS representam um grave problema sanitário que colocam em risco a vida de pacientes, prolongam o tempo de internação e elevam os custos em saúde^(2)^. São medidas de precaução padrão: higienização das mãos, uso de equipamentos de proteção individual (EPIs) adequados ao tipo de exposição, descarte correto de materiais contaminados, aplicação da etiqueta da tosse/higiene respiratória, isolamento de pacientes e limpeza e desinfecção de superfícies^(3)^.

Apesar da relevância da temática, estudos têm demonstrado que o conhecimento de estudantes e profissionais de enfermagem sobre medidas de precaução padrão apresenta fragilidades^(4)^. Assim, percebe-se preocupação de docentes e pesquisadores da área da saúde no que se refere ao ensino da temática da biossegurança, principalmente no contexto atual, em que as formas de aprender e ensinar vêm sofrendo mudanças^(5)^.

Ressalta-se, assim, a necessidade da inclusão de estratégias educacionais que proporcionem aprendizagens significativas, que estejam alinhadas à realidade social e que possam efetivamente promover a incorporação das boas práticas no cuidado em saúde^(6)^. Por esse motivo, destaca-se a contribuição da percepção dos estudantes sobre as diferentes formas de ensino conduzidas, de modo a promover sua participação ativa e incentivar maior engajamento na construção do seu processo de aprendizagem^(7)^.

Nesse contexto, as metodologias ativas transcendem o modelo tradicional de ensino em que o docente transmite o conteúdo e é o centro do saber. Nessa abordagem, o estudante passa a ser o elemento central da aprendizagem, tendo voz ativa e sendo responsável pela busca e análise do conhecimento. Assim, metodologias ativas estimulam o desenvolvimento crítico, criativo e reflexivo dos estudantes de forma individual e coletiva, buscando diferentes formas para solucionar os problemas^(5)^.

Entre as diversas opções de metodologias ativas de ensino, a simulação clínica se destaca como uma estratégia que cria uma situação em ambiente seguro e controlado, no qual os participantes atuam de maneira ativa no processo de aprendizagem, praticando, analisando e tomando decisões a partir da apresentação de uma situação-problema. Dessa forma, a simulação respeita as fases pré-determinadas para a assimilação do conhecimento e norteia o exercício de tomada de decisão^(8)^.

Cada fase elucida aos estudantes o levantamento de problemas, os diagnósticos e a resolutividade. A apropriação do conhecimento é alcançada quando os estudantes expressam seus pensamentos e sentimentos, relatam dúvidas, incertezas e limitações em sua capacidade de agir e de se autoavaliar, aprendendo com suas próprias experiências e com as de seus colegas^(8)^.

A vivência de situações que exijam a aplicabilidade dos conhecimentos em biossegurança dentro de um ambiente seguro, que não traga risco aos pacientes e profissionais/estudantes, pode ser uma forma eficiente de materializar o conhecimento teórico previamente estudado^(6)^. Nessa perspectiva, a simulação como estratégia de aprendizagem possibilita construir conhecimentos, habilidades e atitudes mais consistentes, contribuindo com a qualidade do cuidado de enfermagem^(9)^.

Embora a literatura recente indique benefícios da simulação clínica na formação em saúde, incluindo melhora no conhecimento, na competência clínica e na autoconfiança^(10,11)^, ainda são incipientes os estudos que aprofundem sobre sua aplicação no ensino da biossegurança e, sobretudo, a percepção dos estudantes acerca dessa estratégia pedagógica nesse contexto^(12,13)^. Considerando as fragilidades já evidenciadas no domínio das precauções padrão e no controle de infecções, torna-se relevante compreender se a simulação clínica é uma ferramenta potencial para enfrentar lacunas formativas relacionadas à segurança do paciente e à prática profissional segura.

Diante do exposto, surge a seguinte questão: como os estudantes de enfermagem percebem o uso da simulação clínica como ferramenta de ensino em biossegurança? Nesse contexto, este estudo objetivou compreender a percepção de estudantes de enfermagem acerca da utilização da simulação clínica no ensino de biossegurança.

## MÉTODO

### Aspectos Éticos

Este estudo foi submetido e aprovado pelo Certificado de Apresentação para Apreciação Ética nº 66654622.0.0000.0121 e pelo Comitê de Ética em Pesquisa com Seres Humanos, sob o Parecer nº 6.021.025. Foram respeitadas as diretrizes e normas regulamentadoras da Resolução nº 466/2012 do Conselho Nacional de Saúde.

Os participantes assinaram o Termo de Consentimento Livre e Esclarecido presencialmente antes de iniciar a atividade da pesquisa. A identificação dos participantes feita utilizando a letra inicial “e” seguida de numeração subsequente (e01, e02, e03...), de forma a preservar sua identidade.

### Tipo de Estudo

Trata-se de estudo descritivo de abordagem qualitativa, embasado no referencial teórico-metodológico da *The International Nursing Association for Clinical Simulation and Learning* (INACSL)^(14)^, que traz diretrizes de boas práticas para o desenvolvimento da simulação. Para a organização metodológica, seguiu-se os passos do guia *COnsolidated criteria for REporting Qualitative research* ^(15)^.

### Procedimentos Metodológicos

A aproximação com os estudantes se concretizou por meio de convite direto em sala de aula, no qual o objetivo e a dinâmica da pesquisa foram esclarecidos. Foram incluídos estudantes maiores de 18 anos matriculados na disciplina “Fundamentos de Enfermagem”, ofertada no 3º semestre do curso de graduação em enfermagem, com disponibilidade para participar da simulação no contraturno da disciplina. Ressalta-se que todos os estudantes já haviam participado da aula teórica sobre biossegurança e precauções padrão antes da realização da prática simulada.

Antes da realização da simulação, os participantes responderam a um instrumento de caracterização elaborado pelas pesquisadoras, contendo informações como idade, sexo, experiência prévia com simulação clínica e atuação profissional na área da saúde. Foram excluídos os estudantes que não participaram da aula teórica de biossegurança.

A coleta de dados foi realizada por meio de questionário autoaplicável composto exclusivamente por questões abertas, elaborado para explorar as percepções dos participantes sobre o aprendizado, o conhecimento, a atitude, o raciocínio clínico e o pensamento crítico desenvolvidos a partir da prática de simulação. A opção pelo instrumento autoaplicável visou favorecer a livre expressão das experiências individuais, minimizando as interferências do pesquisador no processo de produção de dados.

### Cenário do Estudo

O laboratório de práticas de simulação do curso de graduação de enfermagem (LABEnf) de uma universidade pública federal da região Sul do Brasil foi o espaço para o desenvolvimento desta pesquisa.

### Coleta e Organização dos Dados

Antes do início da coleta de dados, foi realizado um teste piloto do cenário com estudantes de outras fases do curso de graduação em enfermagem. O objetivo foi verificar a clareza das orientações, o tempo de execução da atividade e a organização dos recursos utilizados. Os dados dessa etapa não foram considerados na análise. O piloto permitiu realizar pequenos ajustes na organização do cenário, sem modificar seus objetivos.

A coleta de dados foi realizada nos meses de abril, maio e agosto de 2023. Para a aplicação do questionário autoaplicável, foi estruturada uma simulação clínica, com imersão dos estudantes para posterior coleta dos dados da pesquisa. A simulação foi organizada de acordo com as diretrizes da INACSL^(14)^, conforme as etapas descritas no Quadro 1.

**Quadro 1:** Etapas de realização da simulação clínica – Florianópolis, SC, Brasil, 2023.

**Planejamento:** A escolha do tema biossegurança baseou-se nas dificuldades identificadas por discentes e docentes durante as atividades teórico-práticas da disciplina “Fundamentos de Enfermagem”. Nessa etapa, foi definida a facilitadora responsável pela condução da simulação.	
**Objetivos de aprendizagem:** **Objetivo geral** Capacitar os estudantes para a utilização adequada dos equipamentos de proteção individual no atendimento ao paciente, considerando as precauções padrão. **Objetivos específicos** Aplicar corretamente os equipamentos de proteção individual conforme as precauções padrão; Identificar riscos de exposição biológica; Adotar medidas de biossegurança durante o atendimento; Realizar condutas adequadas diante de intercorrências clínicas. Os objetivos foram apresentados aos participantes durante o *briefing*.	
**Condutas esperadas dos participantes:** As condutas esperadas correspondem aos comportamentos observáveis utilizados para avaliar o alcance dos objetivos propostos. – Higienização das mãos nos momentos indicados; – Uso adequado de equipamentos de proteção individual (conforme necessário); – Manejo seguro de secreções e fluidos corporais; – Conduta adequada diante da exposição a material biológico; – Comunicação efetiva com o paciente e acompanhante.	
**Estrutura da simulação:** A simulação clínica foi realizada com estudantes organizados em duplas e distribuídos de forma aleatória. Cada dupla participou ativamente da cena. Após a realização da cena, os estudantes assumiam o papel de observadores das duplas subsequentes. Duração das etapas: ***Briefing*:** 10 minutos ***Cena de simulação*:** 15 minutos ***Debriefing*:** 30 minutos	
**Cenário e organização do ambiente** A simulação foi realizada em ambiente estruturado para reproduzir um quarto de clínica médica hospitalar com dois leitos ocupados, possibilitando a vivência de situações simultâneas de cuidado. Cada leito foi equipado com cama hospitalar, roupas de cama e cabeceira em Fowler, ajustada conforme a condição clínica simulada. Mesas auxiliares continham os prontuários com informações clínicas dos pacientes. Os materiais para assistência foram organizados em bancada lateral de fácil acesso. Foram utilizados atores treinados para representar os pacientes, com apoio de dispositivo simulador de braço para punção venosa, ampliando o realismo dos procedimentos. Os equipamentos de proteção individual foram disponibilizados em local visível e acessível, incentivando seu uso conforme as precauções padrão. A organização do ambiente buscou garantir a fidelidade ao contexto clínico, favorecendo a imersão dos participantes e a execução das condutas esperadas.	
**Materiais e recursos:** Foram utilizados materiais compatíveis com o ambiente clínico simulado, incluindo: Equipamentos de proteção individual; Insumos para manejo de material biológico; Materiais para contenção de sangramento; Dispositivo para simulação de punção venosa.	
**Descrição dos casos clínicos**	
**Caso clínico 1:** Maria, 24 anos, foi internada na clínica médica com diagnóstico de pneumonia. Refere que está se sentindo quente e com calafrios desde o início da manhã. A paciente apresentava náuseas e episódios de vômito, mantendo ao seu lado um recipiente com conteúdo gástrico, que foi entregue ao profissional durante o atendimento. Possuía acesso venoso periférico e estava acompanhada da prima.	**Caso clínico 2:** Paula, 22 anos, foi internada na clínica médica com diagnóstico de pielonefrite. Está com acesso venoso periférico, recebendo antibioticoterapia. Tem uma crise renal durante a visita do enfermeiro ao paciente do leito ao lado e, sem querer, puxa o braço e remove o acesso. Inicia-se um sangramento que precisa ser contido. A acompanhante pede ajuda.
***Briefing:*** O *briefing* teve duração aproximada de 10 minutos e foi realizado imediatamente antes do início da simulação. Nessa etapa, os participantes receberam informações gerais sobre a atividade, incluindo objetivos da simulação, contextualização do cenário, tempo de execução e recursos disponíveis no ambiente. Após essa contextualização inicial, foi realizada a leitura padronizada das orientações aos estudantes, conforme descrito a seguir: **Orientações fornecidas aos participantes** Os estudantes foram orientados a assumir o papel de dois enfermeiros responsáveis por pacientes internados em um quarto de clínica médica hospitalar com dois leitos ocupados. Foi informado que ambos haviam acabado de receber o plantão e deveriam iniciar conjuntamente a avaliação dos pacientes. Também foi explicitado que, durante a assistência, poderiam ocorrer intercorrências clínicas, exigindo tomada de decisão, priorização de cuidados e atuação em equipe. Esperava-se que os participantes conduzissem o atendimento de forma colaborativa, assumindo integralmente o cuidado aos pacientes, incluindo avaliação clínica, execução de procedimentos e manejo de possíveis complicações. Foi reforçada a necessidade de adoção de medidas de biossegurança em todas as situações, com uso adequado de equipamentos de proteção individual, especialmente diante do risco de exposição a fluidos corporais. Além disso, os participantes foram informados de que, ao longo da simulação, deveriam: Avaliar as condições clínicas dos pacientes; Identificar as situações de risco; Intervir de forma oportuna; Organizar o cuidado de forma simultânea para ambos os pacientes no mesmo ambiente. Por fim, foi comunicado que a simulação teria duração de 15 minutos, sendo o início e o término sinalizados pela equipe facilitadora. Também foi informado que o cenário disponibilizava todos os materiais necessários para a assistência, incluindo equipamentos de proteção individual, insumos para manejo de secreções, contenção de sangramentos e recursos para punção venosa, podendo ser utilizados conforme o julgamento dos participantes.	
***Debriefing*:** Realizado imediatamente após a simulação, com duração aproximada de 30 minutos, utilizou a técnica *Gather-Analyze-Summarize* ^(5)^. Participaram dessa etapa a facilitadora e uma docente, que atuou como paciente na cena. Foram utilizadas as seguintes questões disparadoras: a) Como você se sentiu durante o desenvolvimento da simulação? b) Como avalia sua atuação durante a cena? c) Quais foram seus principais acertos? d) Quais aspectos poderiam ser aprimorados e como você os melhoraria em uma situação futura?	
**Avaliação:** A avaliação ocorreu de forma integrada ao *debriefing*, por meio de autoavaliação reflexiva dos participantes, considerando os aspectos técnicos, comportamentais e a adoção de medidas de biossegurança. As condutas esperadas previamente definidas foram utilizadas como referência para análise do desempenho, favorecendo a identificação de acertos, lacunas e oportunidades de aprimoramento das competências clínicas.	

A coleta de dados foi realizada por meio de questionário autoaplicável, composto por quatro questões abertas, elaborado com o objetivo de explorar as percepções dos participantes acerca da simulação clínica. O instrumento foi aplicado imediatamente após a realização do *debriefing*. Destaca-se que o tempo destinado ao *debriefing* foi distinto do tempo de resposta ao questionário, sendo este último de aproximadamente 25 a 30 minutos.

### Análise de Dados

Os dados foram organizados em uma planilha do *software Microsoft Office Excel* ^®^, contendo as variáveis de caracterização dos participantes e as respostas às perguntas qualitativas do estudo. Posteriormente, o material textual de análise foi organizado em documento de texto do *Open Office* ^®^, sendo revisado por uma segunda pesquisadora para eliminar erros de digitação ou de ortografia e uniformizar os termos e siglas. Dessa forma, o *corpus* foi submetido à análise por meio do *Interface de R pour les Analyses Multidimensionnelles de Textes et de Questionnaires* (IRAMUTEQ^®^), versão 0.7 alpha 2. Foram processadas as análises de estatísticas textuais clássicas e a Classificação Hierárquica Descendente (CHD), que permite agrupar segmentos de texto em classes lexicalmente homogêneas, a partir da frequência e coocorrência de palavras, representadas em dendrograma, sendo selecionadas as seguintes classes de palavras: adjetivos, substantivos, verbos e formas não reconhecidos pelo dicionário do IRAMUTEQ^®^.

As classes resultantes foram interpretadas qualitativamente à luz da análise de conteúdo^(16)^, articulando os núcleos lexicais identificados aos objetivos do estudo e às percepções expressas pelos participantes. A interpretação das classes foi realizada de forma criteriosa pelos pesquisadores, buscando identificar categorias temáticas representativas e garantir coerência entre os achados lexicais e a discussão dos resultados.

## RESULTADOS

Do total de 65 estudantes matriculados nos dois semestres em que a coleta de dados foi conduzida, 41 atenderam aos critérios de inclusão do estudo, dos quais 26 compareceram à prática de simulação e foram incluídos na pesquisa.

Entre os 26 participantes, houve predomínio de estudantes do sexo feminino (23; 89%). A idade dos estudantes variou entre 20 e 24 anos. Destaca-se que nenhum dos participantes tinha experiência prévia com simulação e também não atuava na área da saúde. Na análise dos dados textuais, o *corpus* total foi formado por 182 segmentos de texto e 3.981 ocorrências, sendo 708 formas distintas. A média de palavras por segmento de texto foi de 22 ocorrências. A análise por estatística textual clássica evidenciou como termos mais frequentes “paciente”, “situação”, “simulação”, “conseguir”, “calma” e “sentir”.

A partir da análise pela CHD, foram identificadas as seguintes classes semânticas: 1) Domínio emocional para a interação e comunicação com o paciente e família; 2) Contribuições da simulação para o aprendizado das práticas de biossegurança; e 3) Potencialidades e fragilidades percebidas pelo estudante sobre sua atuação. A análise por meio da CHD no IRAMUTEQ^®^ evidenciou que as classes 2 e 3 apresentaram relação entre si e estiveram hierarquicamente associadas à classe 1 (Figura 1).

**Figura 1:** Classificação Hierárquica Descendente realizada no *software* IRAMUTEQ^®^ – Florianópolis, SC, Brasil, 2025.

A classe 1 (“Domínio emocional para a interação e comunicação com o paciente e família”) revelou que os participantes identificaram a importância do controle das suas emoções durante a abordagem ao paciente e o impacto dessa regulação emocional sobre a comunicação efetiva com o paciente e acompanhante. Os estudantes perceberam a importância da sua tranquilidade durante o cuidado, o que impactava a confiança sentida pelos pacientes e acompanhantes, fortalecendo o vínculo e contribuindo com a segurança do paciente.

*Acalmar o paciente e acompanhante para que o atendimento seja prestado de forma mais calma, contribuindo com menos erros que podem ocorrer por conta da inquietação de ambos, como deficiência no uso de EPIs* (e14).

*Manter a minha calma para então poder acalmar o paciente e/ou acompanhante* (e11).

*Acalmar o acompanhante e o paciente sem ficar nervosa* (e18).

*Manter a calma ajuda no domínio de ambiente e situação* (e19).

*Percebi a importância da adaptação e do controle emocional na enfermagem* (e19).

Contudo, apesar de reconhecer a importância da comunicação com o paciente e acompanhante e da sua participação ativa durante a simulação, muitos revelaram sentimentos de nervosismo, ansiedade, medo e tensão ao terem que desenvolver a comunicação com o paciente, o que, em muitos momentos, impactou a sua atuação na cena.

*Tentei acalmá-la, mas não consegui. Perguntei quando entrou no hospital e o nível de sua dor, mas somente porque tive ajuda, pois no nervosismo não lembrei* (e05).

*Acalmar o acompanhante e o paciente sem ficar nervosa e entender que tenho que melhorar essa questão de trabalhar sob pressão* (e18).

*Nervosa por ter que conversar com um paciente e não saber responder as perguntas dele* (e19).

Os participantes revelaram sentimentos de empatia e desejo intenso de ajudar o paciente. No entanto, a instabilidade emocional dos estudantes teve como influência comum a preocupação com a paciente.

*Pelo impulso de auxiliar o paciente, não consegui coordenar o atendimento com os protocolos de segurança* (e01).

*Vi o quão perdida eu estava, medo de a paciente ficar com dor* (e04).

*Tentativa de escutar as necessidades do paciente e de ajudá-lo o mais rápido possível* (e09).

*Frustrada e aflita. Um sentimento de impotência para atender os pacientes*, [...] *medo de não conseguir realizar os procedimentos pela falta de prática* (e09).

*Um misto de nervosismo com medo de ser incapaz de realizar os procedimentos necessários junto ao sentimento de querer ajudar a paciente que estava precisando* (e10).

Na classe 2 (“Contribuições da simulação para o aprendizado das práticas de biossegurança”), foi evidenciado que os estudantes perceberam diversas contribuições da prática de simulação clínica para o desenvolvimento de habilidades e competências relacionadas à temática da biossegurança, com destaque à assimilação da técnica de paramentação e ao uso de EPIs, parte fundamental da precaução padrão.

*Essa simulação nos ajuda a compreender sobre os riscos que sofremos e que os pacientes sofrem quando não priorizamos a biossegurança* (e13).

*A partir da simulação, pude observar algumas habilidades práticas minhas para o cotidiano da enfermagem que necessitam de aperfeiçoamento. A utilização da máscara ao entrar em contato com o paciente apresentando sintomas e diagnóstico de infecção respiratória, por exemplo* (e08).

*Contribuiu para eu estar preparada frente a uma situação inesperada, atentando para o que é imprescindível,* [por exemplo] *usar EPIs em uma cena onde necessito atuar rapidamente* (e26).

*Ajudou a relembrar a importância da atenção aos EPIs e o quanto é crucial atentar-se à biossegurança, seja pessoal ou do paciente* (e15).

A partir da prática, os participantes apontaram que a simulação clínica sobre biossegurança proporcionou uma aproximação à experiência cotidiana da prática do enfermeiro e uma preparação mais próxima da realidade, ou seja, do que irão encontrar no campo prático, permitindo um maior contato entre a teoria e a prática. Os estudantes destacaram maior entendimento sobre as técnicas, assimilação do conteúdo teórico, identificação de necessidade de aperfeiçoamento, maior experiência e exposição a situações para a tomada de decisão no que tange às práticas de biossegurança.

*Mostrar um caso real nos faz pensar e visualizar na prática como agir, o que fazer* (e10).

*Agora sei o que preciso melhorar e vejo essa experiência como um passo importante no meu desenvolvimento* (e21).

*A partir do momento que peguei o plantão, eu já imaginei o risco de contaminação e pensei como organizar o trabalho, calçar as luvas, manter a calma, confirmar os dados do paciente, não fazer o uso incorreto de máscaras* (e22).

*Auxiliar no processo de aprendizagem e técnicas de biossegurança essenciais para nossa saúde* (e25).

*Mesmo não tendo conseguido atuar na cena como eu gostaria, acho que serviu de aprendizado. Não repetirei os mesmos erros e não vou esquecer da importância de usar as luvas* (e18).

Além das habilidades técnicas, os estudantes perceberam que a simulação possibilitou, ainda, o estímulo às competências emocionais. Foram revelados sentimentos de superação, autoconfiança e segurança dos estudantes relacionados às técnicas desenvolvidas, fazendo com que reconhecessem a importância da simulação para o aprendizado.

*Me senti pressionada pela situação de duas pacientes precisando da minha ajuda e mal deu tempo da paramentação. Mas vejo que, depois que terminei, fiquei feliz por ter conseguido solucionar o problema* (e06).

*Meu principal destaque foi a execução correta das técnicas seguindo os protocolos estabelecidos, o que me deu mais confiança para atuar em situações reais* (e11).

*A simulação contribui para entender que precisamos manter a calma, que irão acontecer momentos desesperadores, mas nós estudamos para isso e, com calma, iríamos lembrar o que precisamos fazer para ajudar o paciente sem colocar em risco nossa saúde* (e25).

*Foi desafiador, mas muito enriquecedor* (e17).

*Comecei a simulação muito ansiosa e com medo de errar, mas, conforme fui executando as técnicas, minha confiança aumentou* (e26).

Além disso, os estudantes enfatizaram a importância da observação dos colegas durante a realização da cena e do *debriefing* como momento de reflexão para o aprendizado.

*Ver meus colegas ajudou a perceber coisas que eu poderia ter feito, mas que, pela emoção do momento, não me vieram à mente* (e11).

*Contribuiu para pôr em prática nosso conhecimento e ver os colegas fazendo para perceber o que deixamos passar* (e18).

*Só depois que terminou e sentamos na roda que consegui refletir sobre os desafios enfrentados e percebi o quanto essa experiência me ajudou a desenvolver novas habilidades* (e22).

*Auxiliou no sentido de treinar e me sentir à vontade para errar por ser apenas uma simulação. O momento de debriefing ajudou na construção grupal do raciocínio clínico* (e26).

A classe 3 (“Potencialidades e fragilidades percebidas pelo estudante sobre sua atuação”) apresentou que as principais fragilidades percebidas pelos estudantes foram relacionadas às habilidades de comunicação e controle emocional.

*Melhorar a comunicação, utilizar de forma mais adequada os materiais de trabalho, higienização das mãos antes de entrar em contato com os pacientes* (e03).

*Preciso aperfeiçoar minha concentração, meu nervosismo, atenção, ansiedade, além de não ficar tensa com toda a situação* (e13).

*Preciso trabalhar melhor a ansiedade* (e11).

*Foi tranquilo para mim. Acho que consegui fazer o que era solicitado, mas não consegui finalizar a cena porque a minha paciente fez muitas perguntas e eu não soube sair da conversa* (e23).

Alguns participantes revelaram se sentirem tranquilos e confiantes com a realização das técnicas de biossegurança, representando uma importante potencialidade identificada pelos estudantes na sua prática. Isso demonstrou que, quando controlados os aspectos emocionais, o desempenho dos estudantes pode ser influenciado positivamente, impactando a segurança do paciente e do profissional.

*Acredito que um dos pontos fortes da minha atuação foi a paciência e a precaução* [padrão] *ao utilizar luvas, ao ter contato com o sangue da paciente* (e08).

*Durante a simulação, acredito que minha atuação teve vários pontos fortes. Consegui manter a calma e aplicar os conhecimentos adquiridos em sala de aula de forma segura* (e12).

*Consegui aplicar os conhecimentos da sala de aula para a realidade, só que de uma forma mais segura e controlada, que desse condições de testar coisas e receber feedback sem riscos aos pacientes e/ou à gente* (e17).

*Todas as informações estavam passando na minha cabeça, e tive que me concentrar para em meio aos gritos eu ouvir o relato da minha paciente e sempre lembrar de manter a minha segurança e a da paciente* (e02).

*Consegui realizar as técnicas de forma correta seguindo os protocolos, e mantive a calma durante todo o processo* (e11).

Outra potencialidade reconhecida na prática dos estudantes durante a simulação clínica foi a capacidade de trabalhar em equipe. Quando a comunicação aconteceu de forma clara e efetiva, a percepção dos estudantes foi de que o cuidado foi desempenhado com maior organização, agilidade, qualidade e segurança.

*Um dos meus principais pontos fortes foi a capacidade de trabalhar em equipe* (e13).

*Minha atuação foi muito focada na comunicação com a equipe. Tentei manter um bom diálogo com meus colegas para garantir que todos estivessem alinhados*. [...] *mantive uma comunicação clara com meu colega. Isso ajudou a evitar erros e tornou o atendimento mais ágil e organizad*o (e16).

*O meu ponto forte foi ser proativo na comunicação com a minha dupla, garantindo um ambiente colaborativo e organizado* (e20).

*Eu e minha dupla tivemos um pensamento rápido para cada uma se direcionar ao paciente* (e02).

A partir das potencialidades e fragilidades identificadas por meio da simulação clínica, os participantes foram capazes de reconhecer a necessidade de maior aprofundamento no conteúdo teórico-prático, destacando essa questão como um ponto importante a ser melhorado. Por meio da simulação clínica, os estudantes conseguiram identificar quais técnicas relacionadas à biossegurança precisam ser aperfeiçoadas.

*Melhorar a técnica com a ida nas monitorias* (e21).

*Aprofundar mais sobre o que aprendemos na sala de aula* (e24).

*Acho que melhorar a parte da técnica* (e25).

*A agilidade de se paramentar mais rápido e fazer a assepsia correta mesmo que com pouco tempo* (e09).

*A paramentação no geral deve ser feita com mais precisão, além de saber o que usar em cada momento* (e10).

## DISCUSSÃO

A classe 1 evidenciou que a dimensão emocional esteve no centro da experiência dos estudantes. Sentimentos como nervosismo, medo, insegurança e inquietação foram relatados durante a execução das atividades simuladas, especialmente diante da observação de colegas e docentes, aspecto também identificado em investigações anteriores^(17,18)^. Esses achados indicam que a simulação mobiliza reações emocionais semelhantes às do contexto real, aproximando o estudante das exigências do ambiente clínico.

Entretanto, tais emoções não se configuraram apenas como dificuldades, mas como componentes constitutivos do processo de aprendizagem. O ambiente seguro da simulação possibilitou a vivência dessas emoções sem risco ao paciente, permitindo que fossem trabalhadas por meio do *debriefing* estruturado. A reflexão pós-ação favoreceu a compreensão das decisões tomadas, a identificação de falhas e a reorganização de condutas^(19)^, fortalecendo o pensamento crítico e a capacidade de agir com maior segurança.

No contexto da biossegurança, a dimensão emocional assume papel particularmente relevante. A aplicação das precauções padrão exige não apenas o conhecimento técnico, mas também a capacidade de manter condutas seguras diante de situações de pressão ou incerteza. Evidências recentes indicam que níveis elevados de estresse podem interferir na tomada de decisão e no desempenho clínico de estudantes de enfermagem, reforçando a importância do desenvolvimento da autorregulação emocional para a prática segura^(20)^.

A comunicação também se destacou como aspecto essencial da experiência simulada. Os estudantes relataram tanto avanços quanto dificuldades na interação com pacientes, familiares e equipe. A comunicação efetiva constitui elemento central da segurança do paciente, pois viabiliza o compartilhamento claro de informações e a coordenação de ações assistenciais^(21,22)^. Assim, a simulação mostrou-se como um espaço privilegiado para o exercício integrado de habilidades comunicacionais e práticas de biossegurança, ampliando a formação além do domínio técnico.

A classe 2 evidenciou que a simulação clínica contribuiu para a consolidação do aprendizado em biossegurança, especialmente ao favorecer a articulação entre teoria e prática em situações contextualizadas. Além da execução técnica, os estudantes relataram maior percepção dos riscos biológicos envolvidos no cuidado e maior clareza quanto à aplicação das precauções padrão e ao uso adequado de EPIs. O reconhecimento antecipado do risco de contaminação e a organização prévia das condutas demonstram o desenvolvimento de raciocínio clínico orientado à segurança.

Revisões sobre o ensino de biossegurança no Brasil apontam que essa temática ainda é frequentemente abordada de forma fragmentada e centrada em metodologias tradicionais^(23)^. Nesse contexto, a simulação mostrou-se capaz de integrar conhecimento técnico, julgamento clínico e responsabilidade profissional em um mesmo cenário, aproximando o estudante das demandas reais do cuidado.

Os achados deste estudo dialogam com evidências que indicam que intervenções educativas baseadas em simulação favorecem a formação crítico-reflexiva, ao permitir a identificação de erros e o reconhecimento de falhas em ambiente seguro^(24,25)^. Contudo, diferentemente de abordagens exclusivamente expositivas, a experiência simulada exige tomada de decisão sob pressão e mobilização ativa do conhecimento, reduzindo a lacuna entre saber e fazer.

Outro aspecto relevante refere-se à aprendizagem mediada pelo erro. Ao reconhecerem falhas na execução das técnicas e manifestarem intenção de não as repetir, os estudantes demonstraram internalização do aprendizado, reforçando a importância do ambiente seguro para experimentação e reconstrução da prática.

A vivência prévia em ambiente controlado também foi associada ao aumento da autoconfiança e da percepção de preparo para o campo clínico^(26,27)^. Esse resultado é particularmente relevante no ensino da biossegurança, pois a adesão às medidas preventivas não depende apenas do conhecimento dos protocolos, mas da capacidade de aplicá-los com segurança em contextos de urgência e imprevisibilidade. Assim, a simulação contribui para fortalecer a prática segura e a proteção tanto do paciente quanto do profissional.

A classe 3 revelou que a simulação estimulou processos de autoavaliação e reconhecimento de fragilidades. Os estudantes identificaram limitações principalmente relacionadas à comunicação, à tomada de decisão e à execução técnica das práticas de biossegurança, como a paramentação, a assepsia e o controle da ansiedade em situações de pressão. Esse reconhecimento demonstra que a experiência possibilitou olhar crítico sobre a própria atuação.

Esse movimento reflexivo evidencia o papel da simulação como uma estratégia que favorece a autorreflexão e o amadurecimento profissional. A possibilidade de errar em ambiente seguro, receber *feedback* e repensar a conduta contribui para o desenvolvimento de postura mais crítica e responsável^(28)^. No contexto da biossegurança, esse processo é especialmente importante, pois a adesão às medidas preventivas exige não apenas o conhecimento dos protocolos, mas a atenção constante à forma como são aplicados.

A capacidade de trabalhar em equipe também foi percebida como potencialidade relevante. Quando a comunicação ocorreu de forma clara, os estudantes relataram maior organização e agilidade no cuidado. Isso reforça que a prática segura em biossegurança não depende apenas da execução individual correta das técnicas, mas também da articulação entre os membros da equipe, uma vez que intervenções voltadas ao aprimoramento do trabalho em equipe têm sido associadas à melhoria da segurança do paciente e à redução de erros assistenciais^(29)^.

Além disso, a preparação prévia para situações complexas antes da inserção no campo clínico foi apontada como aspecto positivo. A simulação de alta fidelidade permite aproximação com a realidade sem exposição a riscos reais^(17,28)^, favorecendo maior segurança e confiança para uma atuação futura.

Ao considerar conjuntamente as três classes identificadas, observa-se que a simulação clínica contribui para o ensino da biossegurança de forma multidimensional. Além dos conhecimentos adquiridos, a experiência simulada envolve desenvolvimento emocional, aprimoramento da comunicação, consolidação do conhecimento teórico e estímulo à autorreflexão.

Esses resultados reforçam que o ensino da biossegurança, quando mediado por metodologias experiencialmente orientadas, pode favorecer a formação de profissionais mais seguros, críticos e preparados para lidar com situações de risco biológico. Para os docentes, os achados indicam a necessidade de planejar cenários que integrem dimensões técnicas e relacionais, potencializando a formação integral em enfermagem.

### Limitações do Estudo

A limitação deste estudo inclui a realização em uma única instituição de ensino, o que pode restringir a generalização dos resultados para outros contextos formativos.

### Contribuições Para a Área da Enfermagem

Este estudo contribui para o ensino de biossegurança na formação em enfermagem, evidenciando que a simulação clínica favorece não apenas a consolidação do conhecimento técnico sobre precauções padrão, mas também o desenvolvimento de competências emocionais, comunicacionais e reflexivas essenciais para a prática segura.

Os achados indicam que a utilização estruturada de cenários simulados pode preparar estudantes para o ingresso em ambientes clínicos, fortalecendo a autoconfiança, a tomada de decisão e o compromisso com a segurança do paciente. Para os docentes, os resultados reforçam a importância de integrar, no planejamento pedagógico, atividades de simulação associadas a momentos de *debriefing* que promovam reflexão crítica sobre as práticas de biossegurança e sobre o manejo emocional em situações assistenciais.

## CONCLUSÃO

Este estudo permitiu compreender que, na percepção de estudantes de enfermagem, a simulação clínica é uma estratégia relevante para o ensino da biossegurança, pois favorece a integração entre teoria e prática, o fortalecimento da autoconfiança e o desenvolvimento de competências técnico-reflexivas. Os participantes reconheceram que a experiência simulada contribuiu para maior segurança na aplicação das precauções padrão, ao mesmo tempo em que evidenciou fragilidades relacionadas ao domínio emocional e às habilidades de comunicação em situações que exigem tomada de decisão imediata.

As potencialidades identificadas, como o trabalho em equipe e a capacidade de reconhecer pontos a serem aprimorados, indicam que a simulação favorece processos de autoavaliação e amadurecimento profissional. Assim, além do ensino técnico, a simulação clínica se mostrou contributiva para a formação em enfermagem, especialmente no que se refere à prática segura e à segurança do paciente. Recomenda-se que pesquisas futuras investiguem o impacto longitudinal da simulação na adesão às práticas de biossegurança no campo clínico e explorem sua aplicação em diferentes contextos institucionais.

## Data Availability

Todo o conjunto de dados que dá suporte aos resultados deste estudo foi publicado no próprio artigo.

# Translation: Percepción de los estudiantes de enfermería sobre el uso de la simulación clínica en la formación en bioseguridad

**DOI:** 10.1590/1980-220X-REEUSP-2025-0116es

**Authors:** Bruna Pedroso Canever, Saionara Nunes de Oliveira, Caroline Cechinel Peiter, Samara Eliane Rabelo Suplici, Diana Coelho Gomes, Maria Lígia dos Reis Bellaguarda, Diovane Ghignatti da Costa, Aline Lima Pestana Magalhães, Fernanda Marques de Oliveira

**Affiliations:** 1 Universidade Federal de Santa Catarina, Departamento de Enfermagem, Graduação em Enfermagem, Florianópolis, SC, Brasil.; 2 Hospital Universitário Professor Polydoro Ernani de São Thiago, Florianópolis, SC, Brasil.; 3 Unidade Local de Saúde de Santa Maria, Serviço de Pneumologia - Internamento Geral, Lisboa, Portugal.

**Keywords:** Entrenamiento Simulado, Aprendizaje, Seguridad del Paciente, Contención de Riesgos Biológicos, Educación en Enfermería

## Abstract

**Objetivo:**

Comprender las percepciones de los estudiantes de enfermería sobre el uso de la simulación clínica en la educación en bioseguridad.

**Método:**

Estudio cualitativo, exploratorio y descriptivo, desarrollado en 2023, basado en la aplicación de la simulación clínica para el aprendizaje de la bioseguridad. Participaron veintiséis estudiantes del curso “Fundamentos de Enfermería” de una universidad federal del sur de Brasil. Los datos se analizaron utilizando el software IRAMUTEQ^®^.

**Resultados:**

Surgieron tres categorías: (1) Dominio emocional para la interacción y comunicación con el paciente y la familia, evidenciando sentimientos de ansiedad y necesidad de control emocional durante la aplicación de precauciones estándar; (2) Contribuciones de la simulación al aprendizaje de prácticas de bioseguridad, destacando una mayor comprensión del uso de equipos de protección personal, percepción de riesgos y articulación entre teoría y práctica; y (3) Fortalezas y debilidades percibidas por el estudiante respecto a su desempeño, incluyendo la autoevaluación, el reconocimiento de limitaciones técnicas y el fortalecimiento del trabajo en equipo.

**Conclusión:**

La simulación clínica se percibió como una estrategia que favorece el aprendizaje de prácticas de bioseguridad al integrar aspectos técnicos, emocionales y comunicacionales, contribuyendo a una formación más segura y reflexiva.

## INTRODUCCIÓN

El desarrollo de competencias y conocimientos en bioseguridad es fundamental para la formación profesional en enfermería, para el cambio de comportamiento, para la promoción de buenas prácticas en salud y para la seguridad del paciente. Los principios básicos de bioseguridad deben estar bien consolidados para que los estudiantes puedan comprender las situaciones que exigen la aplicación de estos conocimientos a lo largo de su trayectoria formativa. En este sentido, las precauciones estándar son uno de los principios básicos de la bioseguridad, y comprenden el conjunto de medidas que deben aplicarse en la atención a todos los pacientes^(1)^.

Las precauciones estándar deben emplearse en todos los entornos de atención para prevenir las infecciones relacionadas con la atención de salud (IRAS). Las IRAS representan un grave problema de salud que pone en riesgo la vida de los pacientes, prolonga el tiempo de hospitalización y eleva los costos de salud^(2)^. Las precauciones estándar son: higiene de manos, uso de equipo de protección personal (EPP) adecuado al tipo de exposición, desecho correcto de materiales contaminados, aplicación de las normas de tos y higiene respiratoria, aislamiento de pacientes y limpieza y desinfección de superficies^(3)^.

A pesar de la importancia del tema, los estudios han demostrado que los conocimientos de los estudiantes y profesionales de enfermería sobre las precauciones estándar presentan deficiencias^(4)^. Por lo tanto, se observa una preocupación entre los docentes e investigadores del área de la salud en lo que respecta a la enseñanza del tema de la bioseguridad, especialmente en el contexto actual, en el que las formas de aprender y enseñar han estado experimentando cambios^(5)^.

De este modo, se destaca la necesidad de incorporar estrategias educativas que propicien un aprendizaje significativo, que estén alineadas con la realidad social y que puedan promover de manera efectiva la incorporación de buenas prácticas en la atención en salud^(6)^. Por esta razón, se destaca la importancia de la percepción de los estudiantes sobre las diferentes formas de enseñanza implementadas, con el fin de promover su participación activa e incentivar un mayor compromiso en la construcción de su proceso de aprendizaje^(7)^.

En este contexto, las metodologías activas trascienden el modelo tradicional de enseñanza en el que el docente transmite el contenido y es el centro del saber. En este enfoque, el estudiante se convierte en el elemento central del aprendizaje, con voz activa y responsable de la búsqueda y el análisis del conocimiento. Así, las metodologías activas estimulan el desarrollo crítico, creativo y reflexivo de los estudiantes, tanto de manera individual como colectiva, buscando diferentes formas de resolver los problemas^(5)^.

Entre las diversas opciones de metodologías activas de enseñanza, la simulación clínica se destaca como una estrategia que crea una situación en un entorno seguro y controlado, en el que los participantes actúan de manera activa en el proceso de aprendizaje, practicando, analizando y tomando decisiones a partir de la presentación de una situación problemática. De esta manera, la simulación respeta las fases predeterminadas para la asimilación del conocimiento y orienta el ejercicio de la toma de decisiones^(8)^.

Cada etapa aclara a los estudiantes la identificación de problemas, los diagnósticos y la resolución de los mismos. La apropiación del conocimiento se logra cuando los estudiantes expresan sus pensamientos y sentimientos, comparten sus dudas, incertidumbres y limitaciones en su capacidad de actuar y de autoevaluarse, aprendiendo de sus propias experiencias y de las de sus compañeros^(8)^.

La vivencia de situaciones que exijan la aplicación de los conocimientos en bioseguridad dentro de un entorno seguro, que no represente riesgo para los pacientes ni para los profesionales o estudiantes, puede ser una forma eficiente de materializar el conocimiento teórico estudiado previamente^(6)^. Desde esta perspectiva, la simulación como estrategia de aprendizaje permite desarrollar conocimientos, habilidades y actitudes más consistentes, lo que contribuye a la calidad de la atención de enfermería^(9)^.

Aunque la literatura reciente señala los beneficios de la simulación clínica en la formación en salud, incluyendo mejoras en el conocimiento, la competencia clínica y la autoconfianza^(10,11)^, aún son escasos los estudios que profundizan en su aplicación en la enseñanza de la bioseguridad y, sobre todo, en la percepción de los estudiantes respecto a esta estrategia pedagógica en este contexto^(12,13)^. Teniendo en cuenta las deficiencias ya evidenciadas en el ámbito de las precauciones estándar y el control de infecciones, resulta relevante comprender si la simulación clínica es una herramienta potencial para abordar las brechas formativas relacionadas con la seguridad del paciente y la práctica profesional segura.

Ante lo expuesto, surge la siguiente pregunta: ¿cómo perciben los estudiantes de enfermería el uso de la simulación clínica como herramienta de enseñanza en bioseguridad? En este contexto, el objetivo de este estudio fue comprender la percepción de los estudiantes de enfermería sobre el uso de la simulación clínica en la enseñanza de la bioseguridad.

## MÉTODO

### Aspectos Éticos

Este estudio fue presentado y aprobado mediante el Certificado de Presentación para Evaluación Ética n.º 66654622.0.0000.0121 y por el Comité de Ética en Investigación con Seres Humanos, bajo el Dictamen n.º 6.021.025. Se respetaron las directrices y normas reguladoras de la Resolución n.º 466/2012 del Consejo Nacional de Salud.

Los participantes firmaron el Formulario de Consentimiento Libre e Informado en persona antes de iniciar la actividad de investigación. La identificación de los participantes se realizó utilizando la letra inicial “e” seguida de una numeración secuencial (e01, e02, e03...), con el fin de preservar su identidad.

### Tipo de Estudio

Se trata de un estudio descriptivo de enfoque cualitativo, basado en el marco teórico-metodológico de la *Asociación Internacional de Enfermería para la Simulación Clínica y el Aprendizaje* (INACSL)^(14)^, que ofrece directrices de buenas prácticas para el desarrollo de la simulación. Para la organización metodológica, se siguieron los pasos de la guía *COnsolidated criteria for REporting Qualitative research* ^(15)^.

### Procedimientos Metodológicos

El contacto con los estudiantes se llevó a cabo mediante una invitación directa en el salón de clases, en la que se explicaron el objetivo y la dinámica de la investigación. Se incluyeron estudiantes mayores de 18 años matriculados en la asignatura “Fundamentos de Enfermería”, que se imparte en el tercer semestre de la carrera de enfermería, y que tenían disponibilidad para participar en la simulación fuera del horario de clase de la asignatura. Cabe destacar que todos los estudiantes ya habían participado en la clase teórica sobre bioseguridad y precauciones estándar antes de realizar la práctica simulada.

Antes de la simulación, los participantes respondieron un cuestionario de caracterización elaborado por las investigadoras, que contenía información como edad, sexo, experiencia previa con simulación clínica y práctica profesional en el área de la salud. Se excluyó a los estudiantes que no habían participado en la clase teórica sobre bioseguridad.

La recolección de datos se llevó a cabo mediante un cuestionario autoadministrado compuesto exclusivamente por preguntas abiertas, elaborado para explorar las percepciones de los participantes sobre el aprendizaje, el conocimiento, la actitud, el razonamiento clínico y el pensamiento crítico desarrollados a partir de la práctica de simulación. La elección del cuestionario autoadministrado tuvo como objetivo favorecer la libre expresión de las experiencias individuales, minimizando las interferencias del investigador en el proceso de producción de datos.

### Escenario del Estudio

El laboratorio de prácticas de simulación del programa de licenciatura en enfermería (LABEnf) de una universidad pública federal de la región sur de Brasil fue el espacio donde se llevó a cabo esta investigación.

### Recolección y Organización de los Datos

Antes de iniciar la recolección de datos, se realizó una prueba piloto del escenario con estudiantes de otras etapas del programa de licenciatura en enfermería.

El objetivo fue verificar la claridad de las instrucciones, el tiempo de ejecución de la actividad y la organización de los recursos utilizados. Los datos de esta etapa no se tomaron en cuenta en el análisis. La prueba piloto permitió realizar pequeños ajustes en la organización del escenario, sin modificar sus objetivos.

La recolección de datos se llevó a cabo en los meses de abril, mayo y agosto de 2023. Para la aplicación del cuestionario autoadministrado, se estructuró una simulación clínica, con la participación de los estudiantes para la posterior recolección de los datos de la investigación. La simulación se organizó de acuerdo con las directrices de la INACSL^(14)^, siguiendo las etapas descritas en el Cuadro 1.

**Cuadro 1:** Etapas de realización de la simulación clínica – Florianópolis, SC, Brasil, 2023.

**Planificación:** La elección del tema de la bioseguridad se basó en las dificultades identificadas por los estudiantes y los docentes durante las actividades teórico-prácticas de la asignatura “Fundamentos de Enfermería”. En esta etapa, se designó a la facilitadora encargada de dirigir la simulación.	
**Objetivos de aprendizaje:** **Objetivo general:** Capacitar a los estudiantes para el uso adecuado del equipo de protección personal en la atención al paciente, tomando en cuenta las precauciones estándar. **Objetivos específicos:** Aplicar correctamente el equipo de protección personal de acuerdo con las precauciones estándar; Identificar los riesgos de exposición biológica; Adoptar medidas de bioseguridad durante la atención; Adoptar conductas adecuadas ante complicaciones clínicas. Los objetivos se presentaron a los participantes durante el briefing.	
**Conductas esperadas de los participantes**: Las conductas esperadas corresponden a los comportamientos observables que se utilizan para evaluar el logro de los objetivos propuestos. – Higienización de las manos en los momentos indicados; – Uso adecuado del equipo de protección personal (según sea necesario); – Manejo seguro de secreciones y fluidos corporales; – Conducta adecuada ante la exposición a material biológico; – Comunicación efectiva con el paciente y su acompañante.	
**Estructura de la simulación:** La simulación clínica se llevó a cabo con estudiantes organizados en parejas y distribuidos al azar. Cada pareja participó activamente en la escena. Una vez finalizada la escena, los estudiantes asumían el papel de observadores de las parejas siguientes. Duración de las etapas: ***Briefing*:** 10 minutos **Escena de simulación:** 15 minutos ***Debriefing*:** 30 minutos	
**Escenario y organización del entorno** La simulación se llevó a cabo en un entorno diseñado para reproducir una sala de medicina interna de un hospital con dos camas ocupadas, lo que permitió experimentar situaciones simultáneas de atención médica. Cada cama estaba equipada con una cama hospitalaria, ropa de cama y un cabezal en posición de Fowler, ajustado según la condición clínica simulada. Las mesas auxiliares contenían los expedientes médicos con la información clínica de los pacientes. Los materiales para la atención se organizaron en una repisa lateral de fácil acceso. Se utilizaron actores capacitados para representar a los pacientes, con el apoyo de un dispositivo simulador de brazo para la punción venosa, lo que aumentó el realismo de los procedimientos. Los equipos de protección personal se colocaron en un lugar visible y accesible, fomentando su uso de acuerdo con las precauciones estándar. La organización del entorno buscó garantizar la fidelidad al contexto clínico, favoreciendo la inmersión de los participantes y la ejecución de las conductas esperadas.	
**Materiales y recursos:** Se utilizaron materiales compatibles con el entorno clínico simulado, entre ellos: Equipos de protección personal; Suministros para el manejo de material biológico; Materiales para el control de hemorragias; Dispositivo para la simulación de punción venosa.	
**Descripción de los casos clínicos**	
**Caso clínico 1:** María, de 24 años, fue ingresada en la clínica médica con un diagnóstico de neumonía. Refiere que ha estado sintiendo calor y escalofríos desde primera hora de la mañana. La paciente presentaba náuseas y episodios de vómito, y tenía a su lado un recipiente con contenido gástrico, el cual fue entregado al profesional durante la atención. Contaba con un acceso venoso periférico y estaba acompañada por su prima.	**Caso clínico 2:** Paula, de 22 años, fue ingresada en la clínica médica con un diagnóstico de pielonefritis. Cuenta con un acceso venoso periférico y está recibiendo tratamiento con antibióticos. Sufre una crisis renal durante la visita del enfermero al paciente de la cama contigua y, sin querer, tira del brazo y se sale el acceso. Se produce una hemorragia que hay que detener. La acompañante pide ayuda.
***Briefing:*** El *briefing* duró aproximadamente 10 minutos y se llevó a cabo inmediatamente antes del inicio de la simulación. En esta etapa, los participantes recibieron información general sobre la actividad, incluyendo los objetivos de la simulación, la contextualización del escenario, el tiempo de ejecución y los recursos disponibles en el entorno. Tras esta contextualización inicial, se procedió a la lectura estandarizada de las instrucciones para los estudiantes, tal como se describe a continuación: **Instrucciones proporcionadas a los participantes** Se indicó a los estudiantes que asumieran el papel de dos enfermeros a cargo de pacientes hospitalizados en una sala de medicina interna con dos camas ocupadas. Se les informó que ambos acababan de tomar el turno y debían comenzar de manera conjunta la evaluación de los pacientes. También se les explicó que, durante la atención, podrían surgir complicaciones clínicas que exigieran la toma de decisiones, la priorización de los cuidados y el trabajo en equipo. Se esperaba que los participantes llevaran a cabo la atención de manera colaborativa, asumiendo íntegramente el cuidado de los pacientes, incluyendo la evaluación clínica, la realización de procedimientos y el manejo de posibles complicaciones. Se hizo hincapié en la necesidad de adoptar medidas de bioseguridad en todas las situaciones, con el uso adecuado de equipos de protección personal, especialmente ante el riesgo de exposición a fluidos corporales. Además, se informó a los participantes que, a lo largo de la simulación, debían: Evaluar el estado clínico de los pacientes; Identificar las situaciones de riesgo; Intervenir de manera oportuna; Organizar la atención de manera simultánea para ambos pacientes en el mismo entorno. Por último, se les comunicó que la simulación tendría una duración de 15 minutos, y que el equipo facilitador indicaría el inicio y el final. También se les informó que el escenario contaba con todos los materiales necesarios para la atención, incluyendo equipo de protección personal, suministros para el manejo de secreciones, control de hemorragias y recursos para la punción venosa, los cuales podían utilizarse según el criterio de los participantes.	
***Debriefing:*** Se llevó a cabo inmediatamente después de la simulación, con una duración aproximada de 30 minutos, y se utilizó la técnica *Gather-Analyze-Summarize* ^(5)^. En esta etapa participaron la facilitadora y una profesora, quien actuó como paciente en la escena. Se utilizaron las siguientes preguntas de reflexión: a) ¿Cómo te sentiste durante el desarrollo de la simulación? b) ¿Cómo evalúas tu desempeño durante la escena? c) ¿Cuáles fueron tus principales aciertos? d) ¿Qué aspectos podrían mejorarse y cómo los mejorarías en una situación futura?	
**Evaluación:** La evaluación se llevó a cabo de manera integrada al *debriefing*, mediante una autoevaluación reflexiva de los participantes, tomando en cuenta los aspectos técnicos, conductuales y la adopción de medidas de bioseguridad. Las conductas esperadas, definidas previamente, se utilizaron como referencia para el análisis del desempeño, lo que facilitó la identificación de aciertos, carencias y oportunidades para mejorar las competencias clínicas.	

La recolección de datos se llevó a cabo mediante un cuestionario autoadministrado, compuesto por cuatro preguntas abiertas, elaborado con el objetivo de explorar las percepciones de los participantes sobre la simulación clínica. El instrumento se aplicó inmediatamente después de realizar el *debriefing*. Cabe destacar que el tiempo dedicado al *debriefing* fue distinto del tiempo de respuesta al cuestionario, siendo este último de aproximadamente 25 a 30 minutos.

### Análisis de Datos

Los datos se organizaron en una hoja de cálculo del *software Microsoft Office Excel* ^®^, que contenía las variables de caracterización de los participantes y las respuestas a las preguntas cualitativas del estudio. Posteriormente, el material textual para el análisis se organizó en un documento de texto de *Open Office* ^®^, y fue revisado por una segunda investigadora para eliminar errores de digitación u ortografía y uniformar los términos y las siglas. De esta manera, el *corpus* se sometió a análisis mediante la *Interfaz de R para los Análisis Multidimensionales de Textos y Cuestionarios* (IRAMUTEQ^®^), versión 0.7 alpha 2. Se procesaron los análisis estadísticos textuales clásicos y la CJD, que permite agrupar segmentos de texto en clases léxicamente homogéneas, a partir de la frecuencia y la coocurrencia de palabras, representadas en un dendrograma, seleccionándose las siguientes clases de palabras: adjetivos, sustantivos, verbos y formas no reconocidas por el diccionario de IRAMUTEQ^®^.

Las clases resultantes se interpretaron cualitativamente a la luz del análisis de contenido^(16)^, relacionando los núcleos léxicos identificados con los objetivos del estudio y con las percepciones expresadas por los participantes. La interpretación de las clases fue realizada de manera minuciosa por los investigadores, con el fin de identificar categorías temáticas representativas y garantizar la coherencia entre los hallazgos léxicos y la discusión de los resultados.

## RESULTADOS

Del total de 65 estudiantes matriculados en los dos semestres en los que se llevó a cabo la Recolección de datos, 41 cumplieron con los criterios de inclusión del estudio, de los cuales 26 asistieron a la sesión de simulación y fueron incluidos en la investigación.

Entre los 26 participantes, predominaron las estudiantes del sexo femenino (23; 89%). La edad de los estudiantes osciló entre los 20 y los 24 años. Cabe destacar que ninguno de los participantes tenía experiencia previa con la simulación ni trabajaba en el área de la salud. En el análisis de los datos textuales, el *corpus* total estuvo compuesto por 182 segmentos de texto y 3 981 ocurrencias, con 708 formas distintas. El promedio de palabras por segmento de texto fue de 22 ocurrencias. El análisis mediante estadística textual clásica reveló como términos más frecuentes “paciente”, “situación”, “simulación”, “lograr”, “calma” y “sentir”.

A partir del análisis mediante CJD, se identificaron las siguientes clases semánticas: 1) Control emocional para la interacción y la comunicación con el paciente y la familia; 2) Aportes de la simulación al aprendizaje de las prácticas de bioseguridad; y 3) Potencialidades y debilidades percibidas por el estudiante respecto a su desempeño.

El análisis mediante la CJD en el IRAMUTEQ^®^ puso de manifiesto que las clases 2 y 3 presentaban una relación entre sí y estaban jerárquicamente asociadas a la clase 1 (Figura 1).

**Figura 1:** Clasificación jerárquica descendente realizada en el *software* IRAMUTEQ^®^ – Florianópolis, SC, Brasil, 2025.

La clase 1 (“Control emocional para la interacción y la comunicación con el paciente y su familia”) reveló que los participantes identificaron la importancia de controlar sus emociones al interactuar con el paciente y el impacto de esa regulación emocional en la comunicación efectiva con el paciente y su acompañante. Los estudiantes se dieron cuenta de la importancia de mantener la calma durante la atención, lo cual influía en la confianza que sentían los pacientes y sus acompañantes, fortaleciendo el vínculo y contribuyendo a la seguridad del paciente.

*Tranquilizar al paciente y a su acompañante para que la atención se brinde de manera más serena, lo que contribuye a reducir los errores que pueden ocurrir debido a la inquietud de ambos, como el uso incorrecto del equipo de protección personal (EPP)* (e14).

*Mantener la calma para poder tranquilizar al paciente y/o al acompañante* (e11).

*Tranquilizar al acompañante y al paciente sin ponerme nerviosa* (e18).

*Mantener la calma ayuda a controlar el ambiente y la situación* (e19).

*Me di cuenta de la importancia de la adaptación y el control emocional en la enfermería* (e19).

Sin embargo, a pesar de reconocer la importancia de la comunicación con el paciente y el acompañante, así como de su participación activa durante la simulación, muchos revelaron sentimientos de nerviosismo, ansiedad, miedo y tensión al tener que establecer la comunicación con el paciente, lo que, en muchos momentos, afectó su desempeño en la escena.

*Traté de tranquilizarla, pero no lo logré. Le pregunté cuándo ingresó al hospital y cuál era su nivel de dolor, pero solo porque me ayudaron, ya que, debido al nerviosismo, no me acordé* (e05).

*Tranquilizar al acompañante y al paciente sin ponerme nerviosa y entender que tengo que mejorar este aspecto de trabajar bajo presión* (e18).

*Nerviosa por tener que hablar con un paciente y no saber cómo responder a sus preguntas* (e19).

Los participantes revelaron sentimientos de empatía y un intenso deseo de ayudar al paciente. Sin embargo, la inestabilidad emocional de los estudiantes tuvo como factor común la preocupación por la paciente.

*Por el impulso de ayudar a la paciente, no logré coordinar la atención con los protocolos de seguridad* (e01).

*Me di cuenta de lo perdida que estaba, con miedo de que la paciente sintiera dolor* (e04).

*Intento de escuchar las necesidades de la paciente y de ayudarla lo más rápido posible* (e09).

*Frustrada y angustiada. Un sentimiento de impotencia para atender a los pacientes*, [...] *miedo a no poder realizar los procedimientos por falta de práctica* (e09).

*Una mezcla de nerviosismo y miedo a ser incapaz de realizar los procedimientos necesarios, junto con el deseo de ayudar a la paciente que lo necesitaba* (e10).

En la clase 2 (“Aportes de la simulación al aprendizaje de las prácticas de bioseguridad”), se puso de manifiesto que los estudiantes percibieron diversos aportes de la práctica de la simulación clínica al desarrollo de habilidades y competencias relacionadas con el tema de la bioseguridad, destacando la asimilación de la técnica de vestimenta y el uso de EPI, parte fundamental de las precauciones estándar.

*Esta simulación nos ayuda a comprender los riesgos a los que nos exponemos y a los que se exponen los pacientes cuando no priorizamos la bioseguridad* (e13).

*A partir de la simulación, pude observar algunas de mis habilidades prácticas para el día a día de la enfermería que necesitan perfeccionarse. El uso de la mascarilla al entrar en contacto con un paciente que presenta síntomas y un diagnóstico de infección respiratoria, por ejemplo* (e08).

*Me ayudó a estar preparada ante una situación inesperada, prestando atención a lo que es imprescindible,* [por ejemplo] *usar EPI en una situación en la que necesito actuar rápidamente* (e26).

*Me ayudó a recordar la importancia de prestar atención a los EPI y lo crucial que es cuidar la bioseguridad, ya sea la personal o la del paciente* (e15).

A partir de la práctica, los participantes señalaron que la simulación clínica sobre bioseguridad les permitió acercarse a la experiencia cotidiana de la práctica de enfermería y les brindó una preparación más cercana a la realidad, es decir, a lo que encontrarán en el ámbito práctico, lo que facilitó un mayor contacto entre la teoría y la práctica. Los estudiantes destacaron una mayor comprensión de las técnicas, la asimilación del contenido teórico, la identificación de la necesidad de perfeccionamiento, una mayor experiencia y exposición a situaciones para la toma de decisiones en lo que respecta a las prácticas de bioseguridad.

*Mostrar un caso real nos hace pensar y visualizar en la práctica cómo actuar, qué hacer* (e10).

*Ahora sé qué debo mejorar y veo esta experiencia como un paso importante en mi desarrollo* (e21).

*Desde el momento en que me hice cargo del turno, ya imaginé el riesgo de contaminación y pensé en cómo organizar el trabajo, ponerme los guantes, mantener la calma, confirmar los datos del paciente y no usar las cubrebocas de manera incorrecta* (e22).

*Ayudar en el proceso de aprendizaje y las técnicas de bioseguridad esenciales para nuestra salud* (e25).

*Aunque no logré actuar en la escena como me hubiera gustado, creo que sirvió como aprendizaje. No repetiré los mismos errores y no olvidaré la importancia de usar los guantes* (e18).

Además de las habilidades técnicas, los estudiantes percibieron que la simulación también permitió estimular las competencias emocionales. Se manifestaron sentimientos de superación, autoconfianza y seguridad en los estudiantes en relación con las técnicas desarrolladas, lo que les hizo reconocer la importancia de la simulación para el aprendizaje.

*Me sentí presionada por la situación de tener a dos pacientes que necesitaban mi ayuda y apenas tuve tiempo de ponerme el equipo de protección. Pero veo que, una vez que terminé, me sentí feliz de haber podido resolver el problema* (e06).

*Lo que más me llamó la atención fue la ejecución correcta de las técnicas siguiendo los protocolos establecidos, lo que me dio más confianza para actuar en situaciones reales* (e11).

*La simulación ayuda a comprender que debemos mantener la calma, que habrá momentos desesperantes, pero que estudamos para eso y, con calma, recordaremos lo que debemos hacer para ayudar al paciente sin poner en riesgo nuestra salud* (e25).

*Fue un reto, pero muy enriquecedor* (e17).

*Empecé la simulación muy ansiosa y con miedo a equivocarme, pero, a medida que iba aplicando las técnicas, mi confianza fue aumentando* (e26).

Además, los estudiantes destacaron la importancia de observar a sus compañeros durante la representación de la escena y del *debriefing* como momento de reflexión para el aprendizaje.

*Ver a mis compañeros me ayudó a darme cuenta de cosas que podría haber hecho, pero que, por la emoción del momento, no se me ocurrieron* (e11).

*Contribuyó a poner en práctica nuestros conocimientos y a ver a los compañeros en acción para darnos cuenta de lo que se nos pasó por alto* (e18).

*Solo después de que terminó y nos sentamos en círculo pude reflexionar sobre los desafíos que enfrentamos y me di cuenta de cuánto me ayudó esta experiencia a desarrollar nuevas habilidades* (e22).

*Me ayudó en el sentido de practicar y sentirme a gusto para cometer errores, ya que solo era una simulación. El momento de la sesión de reflexión contribuyó a la construcción grupal del razonamiento clínico* (e26).

La clase 3 (“Potencialidades y debilidades percibidas por el estudiante sobre su desempeño”) mostró que las principales debilidades percibidas por los estudiantes estaban relacionadas con las habilidades de comunicación y el control emocional.

*Mejorar la comunicación, utilizar de manera más adecuada los materiales de trabajo, lavarse las manos antes de entrar en contacto con los pacientes* (e03).

*Necesito mejorar mi concentración, mi nerviosismo, mi atención y mi ansiedad, además de no ponerme tensa ante toda la situación* (e13).

*Necesito trabajar mejor la ansiedad* (e11).

*Para mí fue tranquilo. Creo que logré hacer lo que se me pidió, pero no pude terminar la escena porque mi paciente hizo muchas preguntas y no supe cómo salir de la conversación* (e23).

Algunos participantes revelaron sentirse tranquilos y seguros al aplicar las técnicas de bioseguridad, lo que representa un importante potencial identificado por los estudiantes en su práctica. Esto demostró que, cuando se controlan los aspectos emocionales, el desempeño de los estudiantes puede verse influido positivamente, lo que repercute en la seguridad tanto del paciente como del profesional.

*Creo que uno de los puntos fuertes de mi actuación fue la paciencia y la precaución* [patrón] *al usar guantes y al tener contacto con la sangre de la paciente* (e08).

*Durante la simulación, creo que mi desempeño tuvo varios puntos fuertes. Logré mantener la calma y aplicar los conocimientos adquiridos en el salón de clases de manera segura* (e12).

*Logré aplicar los conocimientos del salón de clases a la realidad, pero de una manera más segura y controlada, lo que me permitió probar cosas y recibir feedback sin riesgos para los pacientes ni para nosotros* (e17).

*Toda la información me pasaba por la cabeza, y tuve que concentrarme para, en medio de los gritos, escuchar el relato de mi paciente y recordar siempre mantener mi seguridad y la de ella* (e02).

*Logré realizar las técnicas de manera correcta siguiendo los protocolos, y mantuve la calma durante todo el proceso* (e11).

Otra fortaleza reconocida en la práctica de los estudiantes durante la simulación clínica fue la capacidad de trabajar en equipo. Cuando la comunicación se llevó a cabo de manera clara y efectiva, la percepción de los estudiantes fue que la atención se prestó con mayor organización, agilidad, calidad y seguridad.

*Uno de mis principales puntos fuertes fue la capacidad de trabajar en equipo* (e13).

*Mi desempeño se centró mucho en la comunicación con el equipo. Traté de mantener un buen diálogo con mis colegas para asegurarme de que todos estuviéramos en sintonía*. [...] *Mantuve una comunicación clara con mi colega. Esto ayudó a evitar errores e hizo que la atención fuera más ágil y organizada* (e16).

*Mi punto fuerte fue ser proactivo en la comunicación con mi compañero, garantizando un ambiente colaborativo y organizado* (e20).

*Mi compañero y yo reaccionamos rápidamente para que cada uno se ocupara del paciente* (e02).

A partir de las fortalezas y debilidades identificadas mediante la simulación clínica, los participantes pudieron reconocer la necesidad de profundizar más en el contenido teórico-práctico, destacando este aspecto como un punto importante que debe mejorarse. A través de la simulación clínica, los estudiantes lograron identificar qué técnicas relacionadas con la bioseguridad deben perfeccionarse.

*Mejorar la técnica asistiendo a las sesiones de tutoría* (e21).

*Profundizar más en lo que aprendimos en el salón de clases* (e24).

*Creo que hay que mejorar la parte técnica* (e25).

*La agilidad para vestirse más rápido y realizar la asepsia correctamente, incluso con poco tiempo* (e09).

*En general, el proceso de vestirse debe realizarse con mayor precisión, además de saber qué usar en cada momento* (e10).

## DISCUSIÓN

La clase 1 puso de manifiesto que la dimensión emocional ocupó un lugar central en la experiencia de los estudiantes. Se reportaron sentimientos como nerviosismo, miedo, inseguridad e inquietud durante la realización de las actividades simuladas, especialmente ante la observación de compañeros y docentes, un aspecto también identificado en investigaciones anteriores^(17,18)^. Estos hallazgos indican que la simulación genera reacciones emocionales similares a las del contexto real, acercando al estudiante a las exigencias del entorno clínico.

Sin embargo, dichas emociones no se configuraron únicamente como dificultades, sino como componentes constitutivos del proceso de aprendizaje. El entorno seguro de la simulación permitió experimentar estas emociones sin riesgo para el paciente, lo que facilitó su análisis mediante el *debriefing* estructurado. La reflexión posterior a la acción favoreció la comprensión de las decisiones tomadas, la identificación de fallas y la reorganización de conductas^(19)^, fortaleciendo el pensamiento crítico y la capacidad de actuar con mayor seguridad.

En el contexto de la bioseguridad, la dimensión emocional asume un papel particularmente relevante. La aplicación de las precauciones estándar exige no solo conocimiento técnico, sino también la capacidad de mantener conductas seguras ante situaciones de presión o incertidumbre. Evidencias recientes indican que los niveles elevados de estrés pueden interferir en la toma de decisiones y en el desempeño clínico de los estudiantes de enfermería, lo que refuerza la importancia del desarrollo de la autorregulación emocional para una práctica segura^(20)^.

La comunicación también se destacó como un aspecto esencial de la experiencia simulada. Los estudiantes informaron tanto avances como dificultades en la interacción con pacientes, familiares y el equipo. La comunicación efectiva constituye un elemento central de la seguridad del paciente, ya que permite compartir información de manera clara y coordinar las acciones de atención^(21,22)^. Así, la simulación se reveló como un espacio privilegiado para el ejercicio integrado de habilidades comunicativas y prácticas de bioseguridad, ampliando la formación más allá del ámbito técnico.

La clase 2 puso de manifiesto que la simulación clínica contribuyó a la consolidación del aprendizaje en materia de bioseguridad, especialmente al favorecer la articulación entre la teoría y la práctica en situaciones contextualizadas. Además de la ejecución técnica, los estudiantes informaron una mayor percepción de los riesgos biológicos involucrados en la atención y una mayor claridad respecto a la aplicación de las precauciones estándar y al uso adecuado del EPI. El reconocimiento anticipado del riesgo de contaminación y la organización previa de las conductas demuestran el desarrollo de un razonamiento clínico orientado a la seguridad.

Las revisiones sobre la enseñanza de la bioseguridad en Brasil señalan que este tema aún se aborda con frecuencia de manera fragmentada y centrada en metodologías tradicionales^(23)^. En este contexto, la simulación demostró ser capaz de integrar el conocimiento técnico, el juicio clínico y la responsabilidad profesional en un mismo escenario, acercando al estudiante a las exigencias reales de la atención médica.

Los hallazgos de este estudio concuerdan con la evidencia que indica que las intervenciones educativas basadas en la simulación favorecen la formación crítica-reflexiva, al permitir la identificación de errores y el reconocimiento de fallas en un entorno seguro^(24,25)^. Sin embargo, a diferencia de los enfoques exclusivamente expositivos, la experiencia simulada exige la toma de decisiones bajo presión y la movilización activa del conocimiento, lo que reduce la brecha entre el saber y el hacer.

Otro aspecto relevante se refiere al aprendizaje mediado por el error. Al reconocer fallas en la ejecución de las técnicas y manifestar la intención de no repetirlas, los estudiantes demostraron haber interiorizado el aprendizaje, lo que refuerza la importancia de un entorno seguro para la experimentación y la reconstrucción de la práctica.

La experiencia previa en un entorno controlado también se asoció con un aumento de la autoconfianza y de la percepción de preparación para el ámbito clínico^(26,27)^. Este resultado es particularmente relevante en la enseñanza de la bioseguridad, ya que el cumplimiento de las medidas preventivas no depende solo del conocimiento de los protocolos, sino de la capacidad de aplicarlos con seguridad en contextos de urgencia e imprevisibilidad. Así, la simulación contribuye a fortalecer la práctica segura y la protección tanto del paciente como del profesional.

La clase 3 reveló que la simulación estimuló procesos de autoevaluación y reconocimiento de debilidades. Los estudiantes identificaron limitaciones relacionadas principalmente con la comunicación, la toma de decisiones y la ejecución técnica de las prácticas de bioseguridad, como el uso de indumentaria, la asepsia y el control de la ansiedad en situaciones de presión. Este reconocimiento demuestra que la experiencia permitió una mirada crítica sobre su propia actuación.

Este proceso reflexivo pone de manifiesto el papel de la simulación como una estrategia que favorece la autorreflexión y la maduración profesional. La posibilidad de cometer errores en un entorno seguro, recibir *feedback* y replantearse la conducta contribuye al desarrollo de una postura más crítica y responsable^(28)^. En el contexto de la bioseguridad, este proceso es especialmente importante, ya que el cumplimiento de las medidas preventivas exige no solo el conocimiento de los protocolos, sino también una atención constante a la forma en que se aplican.

La capacidad de trabajar en equipo también se percibió como un potencial relevante. Cuando la comunicación fue clara, los estudiantes informaron de una mayor organización y agilidad en la atención. Esto refuerza la idea de que la práctica segura en materia de bioseguridad no depende únicamente de la ejecución individual correcta de las técnicas, sino también de la coordinación entre los miembros del equipo, ya que las intervenciones orientadas a mejorar el trabajo en equipo se han asociado con una mayor seguridad del paciente y una reducción de los errores de atención^(29)^.

Además, la preparación previa para situaciones complejas antes de la incorporación al ámbito clínico se señaló como un aspecto positivo. La simulación de alta fidelidad permite acercarse a la realidad sin exposición a riesgos reales^(17,28)^, lo que favorece una mayor seguridad y confianza para la práctica futura.

Al considerar conjuntamente las tres categorías identificadas, se observa que la simulación clínica contribuye a la enseñanza de la bioseguridad de manera multidimensional. Además de los conocimientos adquiridos, la experiencia simulada implica desarrollo emocional, mejora de la comunicación, consolidación del conocimiento teórico y estímulo a la autorreflexión.

Estos resultados refuerzan la idea de que la enseñanza de la bioseguridad, cuando se lleva a cabo mediante metodologías orientadas a la experiencia, puede favorecer la formación de profesionales más seguros, críticos y preparados para lidiar con situaciones de riesgo biológico. Para los docentes, los hallazgos indican la necesidad de planificar escenarios que integren dimensiones técnicas y relacionales, potenciando la formación integral en enfermería.

### Limitaciones del Estudio

La limitación de este estudio radica en que se llevó a cabo en una sola institución educativa, lo que puede restringir la generalización de los resultados a otros contextos formativos.

### Aportes al Campo de la Enfermería

Este estudio contribuye a la enseñanza de la bioseguridad en la formación en enfermería, al demostrar que la simulación clínica favorece no solo la consolidación del conocimiento técnico sobre las precauciones estándar, sino también el desarrollo de competencias emocionales, comunicativas y reflexivas esenciales para una práctica segura.

Los hallazgos indican que el uso estructurado de escenarios simulados puede preparar a los estudiantes para su ingreso en entornos clínicos, fortaleciendo la autoconfianza, la toma de decisiones y el compromiso con la seguridad del paciente. Para los docentes, los resultados refuerzan la importancia de integrar, en la planificación pedagógica, actividades de simulación asociadas a momentos de *debriefing* que promuevan la reflexión crítica sobre las prácticas de bioseguridad y sobre el manejo emocional en situaciones de atención.

## CONCLUSIÓN

Este estudio permitió comprender que, en la percepción de los estudiantes de enfermería, la simulación clínica es una estrategia relevante para la enseñanza de la bioseguridad, ya que favorece la integración entre la teoría y la práctica, el fortalecimiento de la autoconfianza y el desarrollo de competencias técnico-reflexivas. Los participantes reconocieron que la experiencia simulada contribuyó a una mayor seguridad en la aplicación de las precauciones estándar, al tiempo que puso de manifiesto debilidades relacionadas con el control emocional y las habilidades de comunicación en situaciones que exigen la toma de decisiones inmediatas.

Las potencialidades identificadas, como el trabajo en equipo y la capacidad de reconocer aspectos que deben mejorarse, indican que la simulación favorece los procesos de autoevaluación y maduración profesional. Así, además de la enseñanza técnica, la simulación clínica demostró ser un elemento contributivo para la formación en enfermería, especialmente en lo que se refiere a la práctica segura y a la seguridad del paciente. Se recomienda que futuras investigaciones analicen el impacto a largo plazo de la simulación en el cumplimiento de las prácticas de bioseguridad en el ámbito clínico y exploren su aplicación en diferentes contextos institucionales.

## Data Availability

Todo el conjunto de datos que apoya los resultados de este estudio fue publicado en el propio artículo.
